# Comprehensive Chemical and Biological Assessment of *Orobanche racemosa* Extracts Using Chromatographic, In Vitro, In Silico, and Cell‐Based Techniques

**DOI:** 10.1002/open.70283

**Published:** 2026-08-17

**Authors:** Nilofar Nilofar, Enver Saka, Mehmet Veysi Cetiz, Maria J. Rodrigues, Eliana Fernandes, Luisa Custodio, Evren Yildiztugay, Ismail Yapıcı, Ilhami Gulcin, Esraa A. Elhawary, Omayma A. Eldahshan, Abdel Nasser B. Singab, Carlos L. Cespedes‐Acuna, Gokhan Zengin

**Affiliations:** ^1^ Department of Biology Science Faculty Selcuk University Konya Turkey; ^2^ Botanic Garden “Giardino dei Semplici” Department of Pharmacy “G. d’Annunzio” University Chieti Italy; ^3^ Department of Medical Biochemistry Faculty of Medicine Harran University Sanliurfa Turkey; ^4^ Centre of Marine Sciences Faculty of Sciences and Technology University of Algarve Faro Portugal; ^5^ Department of Biotechnology Science Faculty Selcuk University Konya Turkey; ^6^ Department of Chemistry Science Faculty Ataturk University Erzurum Turkey; ^7^ Pharmacognosy Department Faculty of Pharmacy Ain Shams University Cairo Egypt; ^8^ Center for Drug Discovery Research and Development Ain Shams University Cairo Egypt; ^9^ Departamento de Ciencias Básicas Facultad de Ciencias Universidad del Bio Bio Chillan Chile

**Keywords:** computational study, cytotoxic effect, enzyme inhibition, natural products, *Orobanche*, orobanchoside

## Abstract

The growing interest in natural product research as a safer and sustainable alternative to synthetic drugs has triggered this study, which evaluates the chemical profile and biological activity of *Orobanche racemosa* extracts. In the current study, extraction was performed from *O. racemosa* using the maceration method with several extraction solvents. The extracts were examined for their antioxidant and enzyme‐inhibitory activities and their effects on cell viability. In high‐performance liquid chromatography–MS/MS analysis, a total of 31 compounds was detected across all studied extracts. The EtOH and EtOH/water extracts consistently exhibited the highest antioxidant capacities in almost all tested assays. Similarly, EtOH and EtOH/water extracts showed vigorous anti‐AChE activity, measuring 2.88 and 2.35 mg/GALAE, respectively. In the cell viability assay, both EA and EtOH extracts of *O. racemosa* demonstrated potent cytotoxicity, significantly reducing viability in normal (HEK293) and cancer cell lines (HepG_2_, SH‐SY5Y), indicating high toxicity without selectivity. Molecular docking screened 384 theoretical compound–target combinations and retained 251 unique complexes with docking scores of −7.0 kcal/mol or lower after duplicated entries were removed. Subsequent single‐trajectory molecular dynamics analysis of seven representative high‐scoring complexes generated hypotheses regarding structural persistence under the simulated conditions; however, these computational predictions require experimental validation.

## Graphical abstract

*Orobanche racemosa* can be considered an effective source of natural bioactive agents in designing functional applications.

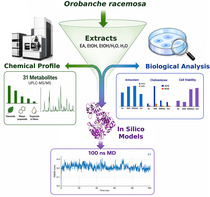

## 1 Introduction

Plants have been used as therapeutic agents for millennia. Despite developments in synthetic chemistry, they remain vital in modern medicine [1]. In 1993, a review by Balandrin et al. stated that natural products represent 50% of all drugs in clinical practice [2]. In developing countries, many people still depend on herbal medicine. For instance, 90% of Africa's population relies on herbal medicine for primary health care [3]. In industrialized countries, such as China, traditional medicine accounts for ≈40% of healthcare, with over 90% of hospitals offering related services [3, 4]. In 2007, ≈38% of adults and 25% of children in the US depended on traditional medicine [5]. Herbal medicine is often used because of its more affordable and nontoxic effects compared to synthetic drugs. Plants have bioactive compounds such as phenols, flavonoids, terpenoids, alkaloids, and tannins [6, 10]. These bioactive compounds are known for their essential medicinal importance, exhibiting antioxidant, anticancer, antimicrobial, antiviral, hepatoprotective, antidiabetic, and antiparasitic properties [11, 15].

Antioxidant enzymes regulate reactive oxygen species (ROS) and reactive nitrogen species (RNS) in the living body [16, 17]. However, an imbalance of ROS and RNS interacts with macromolecules within the cells, such as DNA and RNA, causing mutations or chromosomal abnormalities [18]. These changes disrupt essential processes such as cell cycle regulation and DNA repair, promoting the activation and deactivation of oncogenes and tumor suppression genes, respectively, which lead to cancer development [18, 19]. A wide range of research has been conducted, emphasizing the anticancer effects of plant antioxidants, suggesting that they serve as a promising candidate for developing advancements in cancer treatment strategies [20, 23]. Beyond their antioxidant and anticancer effects, plant bioactive compounds provide extensive other health benefits, such as playing a potential role in managing neurodegenerative disorders [24, 25], slowing the aging process [26, 27], and helping to regulate diabetes [28] by their antienzymatic properties. Altogether, the medicinal importance of plants’ bioactive compounds leads to the development of safer, more effective treatments that support overall health and well‐being.

*Orobanche* is a member of the family Orobanchaceae, the largest genus with more than 100 species. This genus has an annual, biennial, or perennial nonhost‐specific parasitic herb and lacks chlorophyll. These plants are predominantly found in the northern temperate zone [29, 30]. The family Orobanchaceae includes closely related medicinal genera, including *Cistanche*, *Boschniakia*, and *Orobanche* [31]. These plants have some traditional uses to treat malaria, sexual neurasthenia, the nervous system, and gastrointestinal disorders [32]. Recently, many *Orobanche* species have been harvested for folk medicine and functional foods, highlighting their nourishing and therapeutic potential. Nevertheless, a comprehensive study of the pharmacological properties of *Orobanche* species is still lacking.

Although many *Orobanche* species have been used in folk medicine and functional foods for their nourishing and therapeutic properties, a comprehensive pharmacological study of *Orobanche racemosa* remains lacking. In particular, no study to date has combined experimental assays with advanced computational approaches—such as molecular docking and molecular dynamics (MD) simulations—to elucidate the molecular mechanisms underlying the interactions between its bioactive compounds and therapeutic targets. To address this gap, the present study aims to characterize the chemical profiles and evaluate the biological activities, including antioxidant and enzyme‐inhibitory activities and effects on cell viability of several *O. racemosa* extracts, complemented by extensive computational analyses to predict and model the binding affinities and structural stability of its bioactive components with key disease‐related proteins.

## 2 Material and Methods

### 2.1 Plant Collection

The plant specimens were collected in 2022 from a site in the Dedemli (Hadim) area of Turkey's Konya province, at an elevation of 1150 m. Dr Evren Yildiztugay carried out the formal botanical classification of the material. A voucher specimen, under the code EY‐3106, was placed in the Faculty of Science at Selçuk University. Following collection, the aerial parts of the plant were separated immediately and left to dry naturally at room temperature in a shaded, well‐ventilated space. Once fully dried, the plant material was ground into a fine powder. To ensure chemical integrity and prevent deterioration, the powdered samples were sealed in opaque containers and stored under stable conditions.

### 2.2 Plant Extract Preparation

Four solvent systems were employed to isolate bioactive constituents: ethyl acetate, ethanol, 70% ethanol in water, and water. For each extraction, 10 g of the prepared plant material were combined with 200 mL of the respective solvent. The methodology was adapted according to the properties of the solvent. Extractions using organic solvents were performed via maceration for 24 h under ambient conditions. In contrast, the water‐based extraction utilised a 15 min infusion with heated water. Following extraction, the products obtained were concentrated using distinct techniques. The aqueous extract was stabilised by lyophilisation, while the organic solvent fractions were recovered by evaporating the solvents under vacuum using a rotary evaporator.

### 2.3 Total Phenolic and Flavonoid Content

The concentrations of total phenolics and flavonoids within the extracts were quantified using established colorimetric assays, as detailed in the referenced methodology. Calibration curves were prepared using gallic acid (mg GAE/g) and rutin (mg RE/g) as reference standards [33].

### 2.4 UHPLC/MS/MS Analysis

The phytochemical composition of *O. racemosa* extracts was evaluated employing the previously described method, involving ultra high‐performance liquid chromatography (UHPLC) coupled with an electrospray ionization tandem mass spectrometry (ESI‐MS/MS) detector [34]. The analytical details are given in the supplemental materials.

### 2.5 Antioxidant Assays

The antioxidant capacity was evaluated using a series of in vitro tests based on the experimental framework of a previous study [34]. The extracts were screened for reducing power using the FRAP and CUPRAC assays, and for radical scavenging activity using the DPPH and ABTS assays. The results of these four assays were quantified and standardised against the antioxidant Trolox and expressed as milligrams of Trolox equivalent per gram of dried extract (mg TE/g). Additionally, total antioxidant capacity was measured using the phosphomolybdenum method (PBD assay), with results expressed in millimoles of Trolox equivalent per gram (mmol TE/g). Finally, the ability of the extracts to bind metal ions was evaluated using a metal chelation assay, with activity reported as milligrams of EDTA equivalent per gram of extract (mg EDTAE/g).

### 2.6 Enzyme Inhibitory Assays

The inhibitory profiles of the enzymes in the extracts were evaluated against five key enzymes—acetylcholinesterase (AChE), butyrylcholinesterase (BChE), tyrosinase, amylase, and glucosidase—using well‐documented colourimetric procedures [34]. To standardise the results, inhibition was quantified using established reference compounds. Cholinesterase inhibition was expressed as milligrams of galantamine equivalent per gram of extract (mg GALAE/g). Amylase and glucosidase activity was expressed in milligrams of acarbose equivalent per gram (mg ACAE/g), and tyrosinase inhibition was reported in milligrams of kojic acid equivalent per gram (mg KAE/g). Additionally, in line with a complementary line of investigation, the extracts were analyzed for their inhibitory effects on human carbonic anhydrase I and II (hCA I and II) isoenzymes.

### 2.7 Cell Culture and Cell Viability Assessment

Human hepatoblastoma HepG2 (RRID: CVCL_0027), human neuroblastoma SH‐SY5Y (RRID: CVCL_0019), and human embryonic kidney HEK293 (RRID: CVCL_0045) cell lines were kindly provided by the Center for Biomedical Research (CBMR), University of Algarve, Portugal. Cell lines were cultured following established protocols [35]. For viability testing, cells were distributed into 96‐well plates at 5000 cells per well and allowed to adhere overnight. The cultures were then treated for 72 h with the test extracts at a concentration of 100 μg/mL. A separate group of cells receiving only 0.5% DMSO functioned as the untreated control. Following the incubation period, cellular viability was quantified via the MTT assay, based on the reduction of 3‐(4,5‐dimethylthiazol‐2‐yl)‐2,5‐diphenyltetrazolium bromide, in accordance with the earlier method [35]. Results are presented as a percentage of viability relative to the DMSO‐treated control group.

### 2.8 Computational Study

#### 2.8.1 Molecular Docking

To this end, extensive molecular docking analyses were performed to evaluate the binding affinities of key phytochemicals obtained from *O. racemosa* against a broad spectrum of biologically important targets. The list of enzymes included major metabolic enzymes such as *α*‐glucosidase (7KBJ), *α*‐amylase (2QV4), tyrosinase (6QXD), BChE (6EQP), and AChE (7E3H). It also included regulatory and signaling proteins that were highly expressed in hepatic cell lines [36], namely AKT (4GV1), BRPF1 (5MWZ), CDK2 (6GUE), CDK4 (2W96), Cyclin D1 (2W99), MYC (1NKP), PPARγ (1I7I), PD‐L1 (5N2F), and TERT (5CQG; nonhuman) [37, 40]. Given their established relevance in neurodegenerative disorders, SH‐SY5Y neuroblastoma cells were selected as a reference model for proteins implicated in apoptosis, oxidative stress, inflammation, and cellular signaling. The present study focused on intrinsic apoptosis regulators Bax (3PL7) and BCL2 (6O0K), executioner caspase‐3 (3GJQ) and initiator caspase‐9 (1JXQ), oxidative stress modulator Keap1 (6T7V), inflammatory transcription factor NFκB (4FA6), proliferation‐associated GTPase Nras (8VM2), and stress‐activated kinase p38 MAPK (1A9U). These targets offer significant insights into the molecular mechanisms underlying neurodegenerative processes in SH‐SY5Y cells. Docking simulations were executed using AutoDock Vina v1.1.2 with an exhaustiveness parameter of 32. The potential binding pockets were predicted, and grid box coordinates were defined using POCASA v1.1, as detailed in Table S1 [41]. The validation of the docking protocol was achieved through the use of native ligand redocking and root mean square deviation (RMSD) calculations [42, 43]. A thorough examination of protein–ligand interactions was conducted, encompassing various noncovalent interactions such as hydrogen bonds, hydrophobic contacts, and *π*‐interactions. The protein–ligand interaction profiler was utilized as a tool to facilitate this analysis [44, 45]. Visualization of binding poses and interaction networks was performed using PyMOL v2.5.8.

#### 2.8.2 MD Simulations

The dynamic properties and stability of the highest‐affinity protein–ligand complexes were examined using MD simulations in GROMACS 2024 [46]. he selected systems comprised the top‐ranked docked phytochemical‐protein pairs: leucocceptoside A with BChE, verbascoside with BChE, verbascoside with AKT, leucocceptoside A with AKT, verbascoside with PD‐L1, verbascoside with Keap1, and eriodictyol hexoside with Keap1 (Table S2). All simulation systems were prepared via the CHARMM‐GUI web server [47, 48] using the CHARMM36m force field for proteins and the CHARMM General Force Field for ligands [49]. The complexes were solvated in a cubic TIP3P water box with a minimum 10 Å distance from any solute atom to the box edges and neutralized with Na^+^ and Cl^−^ ions at a physiological concentration of 0.15 M. Energy minimization was carried out using the steepest descent method until the maximum force on the system dropped below 1000 kJ·mol^−1^·nm^−1^. Two equilibration steps followed: 500 ps under constant volume and temperature and 1 ns under constant pressure and temperature at 310 K. All covalent bonds were constrained using the LINCS algorithm. Long‐range electrostatic interactions were computed via the Particle Mesh Ewald approach with a 12 Å real‐space cutoff, and short‐range nonbonded forces were treated with the Verlet cutoff scheme. Finally, 100 ns production runs were performed for each complex to assess conformational fluctuations, interaction persistence, and overall binding stability throughout the simulations.

#### 2.8.3 Statistical Analysis

All experiments were conducted three times. Data from the chemical‐content, antioxidant, and enzyme‐inhibition assays were analyzed using one‐way ANOVA followed by Tukey's multiple‐comparisons test. Cell‐viability data were analyzed using two‐way ANOVA, with extract and cell line as factors, followed by Tukey's multiple‐comparisons test. Analyses were performed using GraphPad Prism v9.2, and *p* < 0.05 was considered statistically significant.

## 3 Results and Discussion

### 3.1 Total Phenolic and Flavonoid Content

Plant secondary metabolites (phenolic compounds and flavonoids) are the main bioactive compounds that possess potent antioxidant properties, which are beneficial for health [50]. Among more than 100 species of *Orobanche*, only a few have been studied by phytochemical methods. Recent studies reported high yields of phenolic compounds from *Orobanche cernua* and *O. coerulescens* [30, 51], but no study has reported the phytochemical profile of *O. racemosa*. This study investigated the total phenolic and flavonoid contents of *O. racemosa*, and the results are illustrated in Table 1. Among them, EtOH exhibited the highest values for TPC and TFC, measuring 85.73 (mg GAE/g) and 7.05 mg RE/g, respectively, indicating that it was the most effective solvent for extracting phenolic and flavonoid compounds from *O. racemosa*. Closely followed by EtOH/water extracts measuring 81.85 mg GAE/g and 6.64 mg RE/g, respectively,making it the second most efficient solvent. Multiple studies [52, 53], including our various previous studies [54, 55], have demonstrated that ethanol and hydro‐ethanol extract phenolic compounds from natural products more effectively. Compared with the other extracts studied, EA extracts had the lowest TPC and the second‐highest TFC, suggesting they were the least effective at extracting phenolic compounds; however, they had the highest ability to extract flavonoids. Overall, the results indicated that the choice of solvents significantly impacted the extraction efficiency of phenolic and flavonoid compounds. Compared with other *Orobanche* species, these values fall within a generally similar, albeit somewhat lower, range. For instance, in an earlier study by Abes et al. [56], the total phenolic content in extracts from *O. foetida* and *Orobanche crenata* ranged from 3.02 to 19.99 mg GAE/g. Bedoui et al. [57] also reported that the total phenolic and flavonoid contents in *O. foetida* extract were 23.13 mg GAE/g and 19.31 mg quercetin equivalent (QE)/g, respectively. In another study by Attia et al. [58], the total phenolic and flavonoid contents in *O. crenata* and *O. lavandulacea* were found to be 0.50–3.53 mg GAE/g and 0.02–6.78 mg catechin equivalent (CE)/g, respectively. Taken together, the current study found that *O. racemosa* contained remarkable levels of total bioactive components compared with other members of the genus *Orobanche*.

**TABLE 1:** Total phenolic and flavonoid contents and radical scavenging effects of the tested extracts.

Extracts	TPC, mg GAE/g	TFC, mg RE/g	DPPH, mg TE/g	ABTS, mg TE/g
EA	49.00 ± 0.33^d^	6.69 ± 0.08^b^	340.81 ± 11.40^d^	371.71 ± 3.00^d^
EtOH	85.73 ± 0.84^a^	7.05 ± 0.06^a^	514.44 ± 8.48^a^	597.09 ± 2.16^a^
EtOH/water	81.85 ± 0.79^b^	6.64 ± 0.14^b^	479.72 ± 8.56^b^	538.71 ± 5.22^b^
Water	76.61 ± 0.90^c^	5.48 ± 0.06^c^	380.57 ± 4.90^c^	423.62 ± 1.18^c^

*Note*: Values are reported as mean ± SD of three parallel measurements.

Abbreviations: GAE: gallic acid equivalents; RE: rutin equivalents; and TE: trolox equivalents. Different letters indicate significant differences between the tested extracts (*p* < 0.05).

### 3.2 UPLC/MS/MS Tentative Identification of *O. racemosa* Metabolites

**FIGURE 1:**
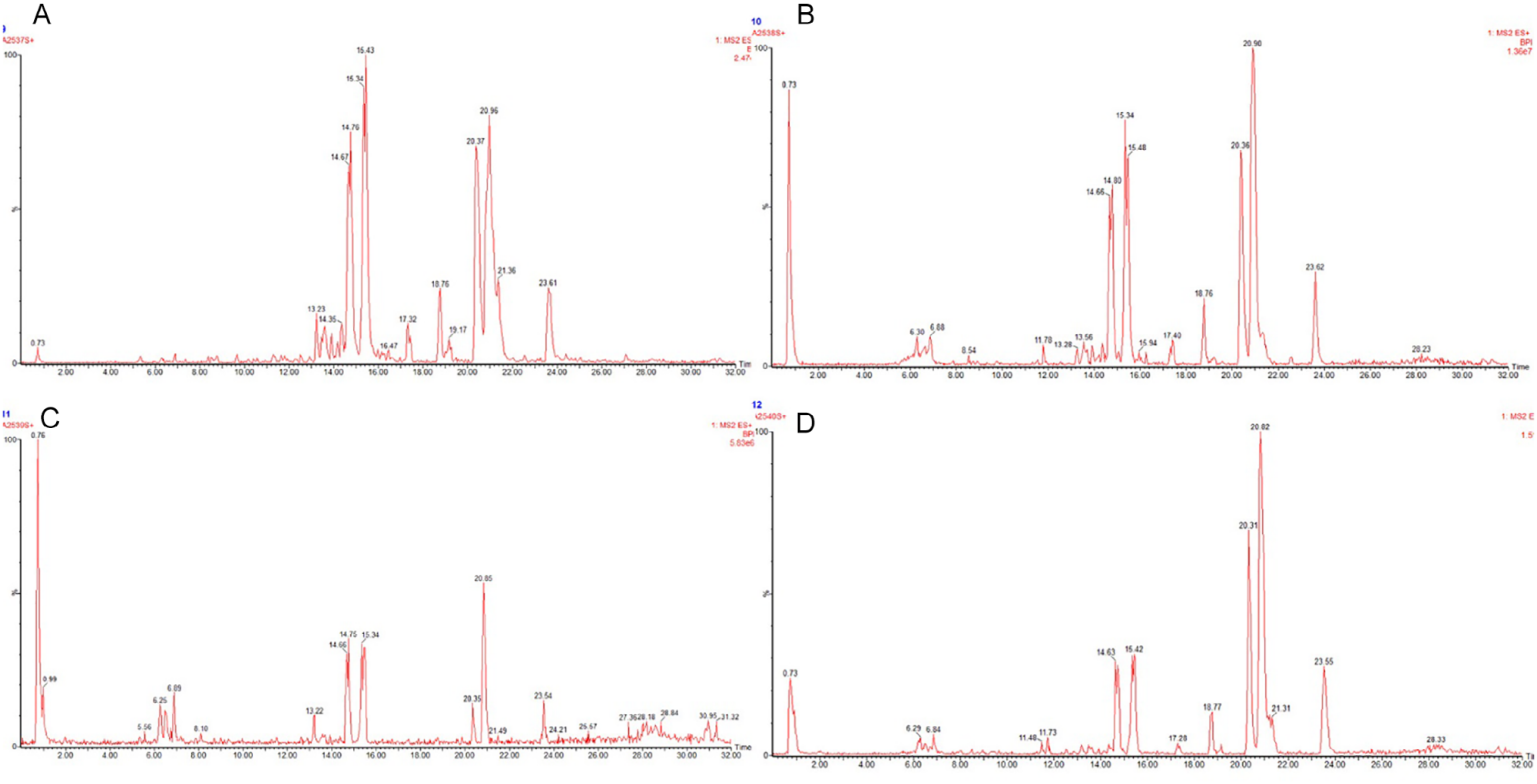
BPI chromatograms in ESI positive ion mode for (A) ethyl acetate, (B) ethanol, (C) ethanol/water, and (D) water.

**FIGURE 2:**
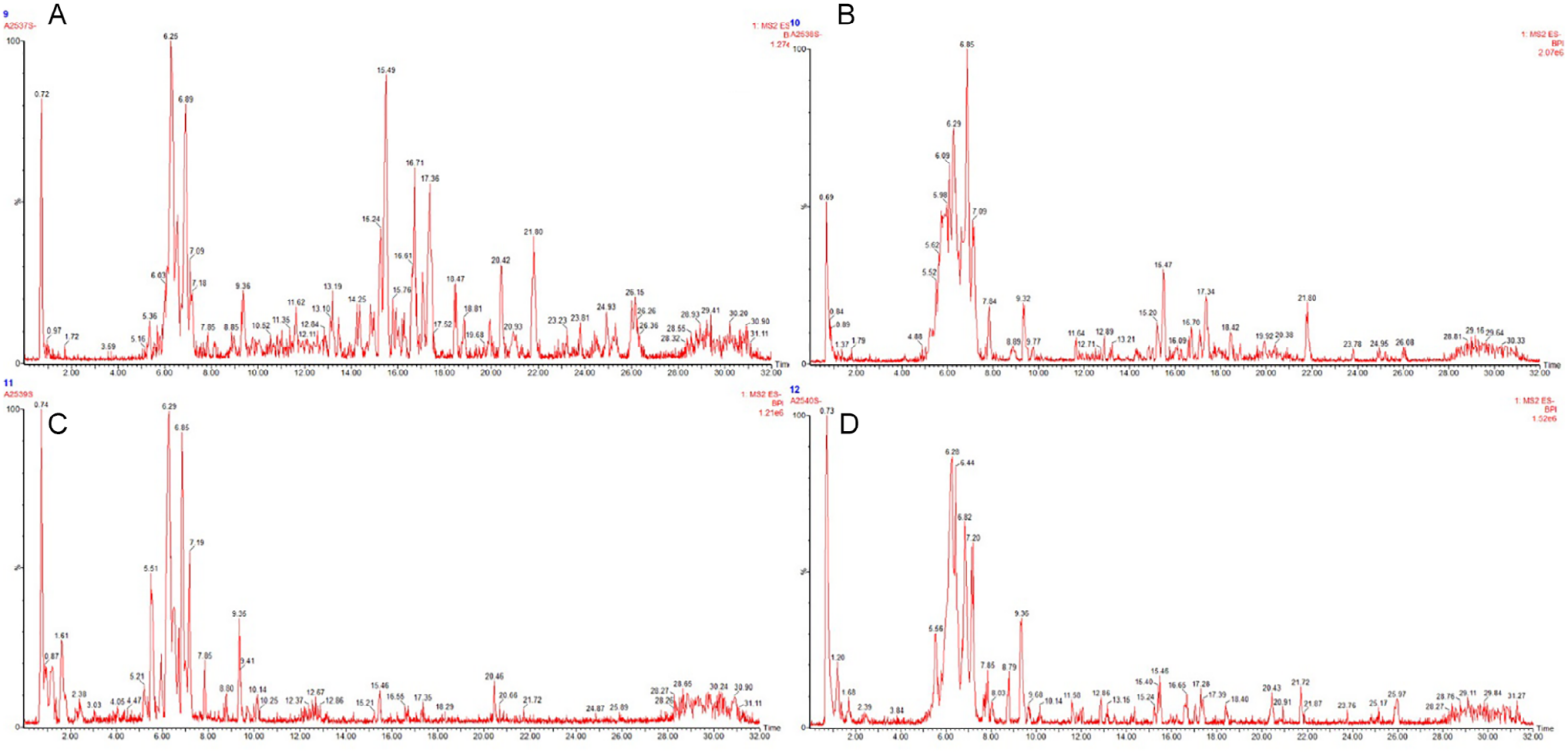
BPI chromatograms in ESI negative ion mode for (A) ethyl acetate, (B) ethanol, (C) ethanol/water, and (D) water.

**FIGURE 3:**
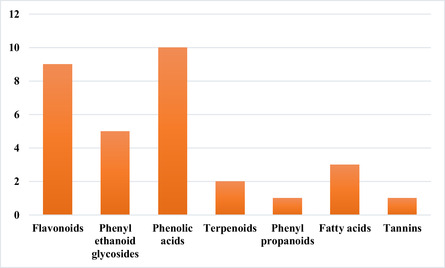
Phytochemical classes of the tentatively identified metabolites from *Orobanche racemose*.

In this study, four extracts from *O. racemosa*, a plant native to the Turkish flora, were comparatively profiled using UPLC/MS^n^ analysis in ESI positive and negative ion modes (Figures 1 and 2). The tentative identification revealed the presence of thirty‐one components mainly as flavonoids, phenolic acids, phenyl propanoids, phenyl ethanoids, terpenoids, fatty acids, and tannins (Figure 3). Weeds thrive in agricultural environments. However, in certain areas of the world, they are consumed by humans as food, and they can represent a source of valuable active ingredients of ethno‐medical interest. Herein, thirty‐one tentatively identified metabolites were detected from four extracts of *O. racemosa*. The most abundant metabolites were from the flavonoids, phenolic acids, and phenyl ethanoids phytochemical classes. Certain extracts showed a higher number of identified components, where the ethyl acetate came first, followed by the aqueous, ethanol/water, and the ethanol extract in a descending manner. Phenyl ethanoids and phenolic acids were highly accumulated in both the ethanol/water and the aqueous extracts, where fatty acids were prominently found in the ethyl acetate. Likewise, phenyl propanoids were spatially present in the aqueous and ethanol/water extracts. The aforementioned phytochemical distribution between the four extracts had a certain impact on their observed biological activities.

#### 3.2.1 Flavonoids

Nine flavonoids and their derivatives were tentatively detected, with some of them reported before from the genus Orobanche (marked with * in Table 2). A deprotonated molecular ion peak was observed at *m/z* 591 with molecular formula of C_27_H_34_O_15_ and its daughter peaks appeared at *m/z* 457, 384, 331, 297, 287, 231, 181, and 159 where the peak at *m/z* 297 is for the aglycone methyl ether moiety while that at *m/z* 181 is due to loss of one hexose moiety; thus, this peak was tentatively identified as apigenin‐methyl ether‐hexoside‐pentoside [68]. The aglycone peak for the aforementioned compound was shown at *m/z* 269, confirming the apigenin aglycone [59, 63, 72]. Another flavonoid glycoside was detected with a parent peak at *m/z* 449 and MS/MS fragments at *m/z* 419, 384, 339, 299, 257, 239, 197, 175, 157, and 151, where the characteristic fragments at *m/z* 197 and 151 confirmed the compound identity as eriodictyol‐hexoside [70, 71]. Methyl‐retusin, an isoflavonoid, presented a deprotonated peak at *m/z* 297 with fragments at *m/z* 267, 232, 179, and 118, where the fragment at *m/z* 267 is a reported characteristic fragment for this compound [69]. Moreover, a deprotonated peak was detected at *m/z* 505 with fragments at *m/z* 423, 410, 339, 325, 317, 301, 293, and 271, which was tentatively assigned to quercetin‐acetyl hexoside [75]. In addition, a parent peak was detected at *m/z* 461 with its aglycone fragment at *m/z* 285, which confirmed the compound identity as luteolin‐hexouronide, which was previously reported from the genus Orobanche [76].

**TABLE 2:** Tentatively identified metabolites from *Orobanche racemosa* extracts.

No.	Compound name	**Molecular** **formula**	**[M‐H]** ^**−**^ ***m/z***	**MS/MS** **fragments**	Composition				Ref.
					**9** **Eth. Ac.**	**10** **Ethanol**	**11** **Ethanol/water**	**12** **Aqueous**	
**Phenyl ethanoid glycosides**									
1	Verbascoside^a^	C_29_H_36_O_15_	623	461, 434, 319, 311, 1181, 167	√	√	√	√	[59, 61]
2	Leucosceptoside^a^	C_30_H_38_O_15_	637	573, 531, 487, 473, 430, 401, 338, 318, 311, 256, 241, 206	—	—	√	—	[60]
3	Acteoside^a^	C_29_H_36_O_15_	623	526, 437, 374, 327, 299, 215, 200, 179	√	√	√	√	[60]
4	Acetyl‐poliumoside^a^	C_36_H_49_O_19_	665	607, 479, 453, 373, 339, 329, 187	√	√	√	√	[60]
5	Acetyl‐poliumoside isomer^a^	C_36_H_49_O_19_	665	622, 599, 551, 532, 475, 445, 363, 267, 205, 181	—	—	√	√	[60]
**Phenolic acids**									
6	Quinic acid^a^	C_7_H_12_O_6_	191	181, 159, 127, 111	—	—	√	√	[57]
7	Caffeic acid pentosyl hexouronide^a^	C_20_H_30_O_14_	487	430, 356, 318, 292, 197, 175	√	—	√	√	[57]
8	*P*‐hydroxy benzoic acid rutinoside	C_19_H_26_O_12_	445	381, 373, 361, 337, 327, 311, 307, 175, 150	—	—	√	√	[62]
9	Vanillic acid hexoside^a^	C_14_H_18_O_9_	329	314, 258, 254, 227, 215, 164	—	√	√	√	[57]
10	Caffeoyl malic acid^a^	C_13_H_12_O_8_	295	291, 275, 253, 179	√	√	√	√	[63]
11	Caffeic acid derivative	—	357	353, 313, 298, 271, 235, 179, 187	√	√	—	—	[64]
12	Sinapoyl hexose^a^	C_17_H_22_O_10_	385	339, 326, 299, 275, 229, 203, 181	—	√	√	√	[57]
13	Acetyl‐caffeoyl quinic acid	C_17_H_21_O_9_	395	337, 327, 311, 245, 237, 181, 179	√	—	—	—	[65]
14	Ferulic acid derivative^a^	—	459	454, 396, 352, 312, 284, 237	√	—	—	√	[63]
15	Protocatechuic acid pentosyl hexoside^a^	C_18_H_28_O_14_	461	454, 353, 301, 296, 255, 232, 184	√	—	—	—	[63]
**Terpenoids**									
16	Oleanolic acid‐hexouronide	C_36_H_54_O_9_	629	501, 461, 446, 433, 381, 315, 282, 227, 193, 165	—	—	√	—	[66]
17	Lactinolide hexoside	C_16_H_28_O_10_	361	310, 293, 258, 247, 222, 185, 161	√	—	—	—	[67]
**Phenyl propanoids**									
18	Orobanchoside^a^	C_29_H_36_O_16_	639	350, 179, 161, 113	—	—	√	√	[61]
**Flavonoids**									
19	Apigenin‐methyl ether‐hexoside‐pentoside	C_27_H_34_O_15_	591	457, 384, 331, 297, 287, 231, 181, 159	√	√	√	√	[68]
20	Tetrahydroxyflavanone	—	287	264, 231, 212, 179	—	—	—	√	[69]
21	Eriodictyol‐hexoside^a^	C_21_H_22_O_11_	449	419, 384, 339, 299, 257, 239, 197, 175, 157, 151	—	—	√	—	[70, 71]
22	Apigenin^a^	C_15_H_10_O_5_	269	252, 227, 214, 186, 146	√	√	—	—	[59, 63, 72]
23	Epicatechin	C_15_H_14_O_6_	291	285, 267, 253, 181, 151, 135	√	—	—	—	[73]
24	8‐methyl‐retusin	C_17_H_14_O_5_	297	267, 232, 179, 118	—	√	—	√	[69]
25	*Dihydroxy*‐trimethoxy flavone	C_18_H_16_O_7_	343	331, 311, 296, 267, 249, 225, 181	√	√	—	√	[74]
26	Quercetin‐acetyl hexoside	C_23_H_22_O_13_	505	423, 410, 339, 325, 317, 301, 293, 271	√	—	—	—	[75]
27	Luteolin‐hexournide^a^	C_21_H_18_O_12_	461	436, 403, 369, 354, 341, 339, 311, 285	√	—	—	√	[76]
**Fatty acids**									
28	9‐oxo‐Octadecadienoic acid	C_18_H_30_O_3_	293	279, 264, 237, 225, 187, 161	√	√	—	√	[64]
29	*n*‐Butyl palmitate^a^	C_20_H_40_O_2_	311	299, 290, 239, 195, 191	√	—	—	—	[59]
30	*n*‐Butyl palmitate isomer^a^	C_20_H_40_O_2_	311	293, 253, 239, 181, 171	√	—	—	—	[59]
**Tannins**									
31	Hydroxy‐gallic acid derivative	—	313	271, 263, 174, 160, 150	√	√	—	√	[77]
No. of identified compounds					20	13	15	19	—

a For the metabolites previously identified from genus *Orobanche*.

#### 3.2.2 Phenyl Ethanoid Glycosides

Phenyl ethanoids represent a special class of phytoconstituents that have been reported from the genus *Orobanche*. Five peaks were detected belonging to this class in our study, including verbascoside (*m/z* 623) [59, 61], leucosceptoside (*m/z* 637) [60], acteoside (*m/z* 623) [60], 2‐acetyl‐poliumoside and its isomer (*m/z* 665) [60]. The fragmentation patterns and molecular formulas for the aforementioned compounds were listed in Table 2.

#### 3.2.3 Phenolic Acids

Phenolic acids represented one of the main classes reported from the genus *Orobanche*. Herein, 10 components were tentativelydetected and classified as phenolic acids or their derivatives. A peak was traced at *m/z* 191 with fragments at *m/z* 181, 159, 127, and 111, and was tentatively identified as the famous phenolic acid, quinic acid [57]. Besides, four caffeic acid derivatives were also detected, showing caffeoyl fragment at *m/z* 179, and they were tentatively identified as caffeic acid pentosyl hexouronide (*m/z* 487) [57], caffeoyl malic acid (*m/z* 295) [63], caffeic acid derivative (*m/z* 357) [64], and acetyl‐caffeoyl quinic acid (*m/z* 395) [65]. Other examples of phenolic acid components were also detected in our study and were listed in Table 2.

#### 3.2.4 Terpenoids

Two triterpenoid peaks were detected in our study and were tentatively assigned to oleanolic acid‐hexouronide (*m/z* 629) [66] and lactinolide hexoside (*m/z* 361) [67].

#### 3.2.5 Phenyl Propanoids

A deprotonated major peak was observed at *m/z* 639 with MS/MS fragments at *m/z* 350, 179, 161, and 113 due to the successive loss of a caffeoyl moiety (*m/z* 179), followed by water loss (*m/z* 161), which was tentatively identified as orobanchoside [61].

#### 3.2.6 Fatty Acids

Three fatty acids were present in the tentative identification and were detected at *m/z* 293 and *m/z* 311, which were tentatively defined as 9‐oxo‐octadecadienoic acid [64], *n*‐butyl palmitate, and its isomer [59].

#### 3.2.7 Tannins

One tannin peak was detected at *m/z* 313 with fragments at *m/z* 271, 263, 174, 160, and 150, which was assigned to hydroxy‐gallic acid derivative [77].

Genus *Orobanche* represents a well‐documented edible part of the plant kingdom with reported phytochemical composition of flavonoids, alkaloids, lignans, phenylpropanoids, phenylethanoids, and others [30]. Previous literature reports had shown the phytochemical richness of this genus such as the total polyphenol and tannin contents from *O. crenata* and *O. foetida* extracts ranged from 3.02–19.99 mg GAE/g DW for polyphenol contents and from 0.09 to 0.32 mg EC/g DW for tannin contents [56]. Moreover, the *n*‐hexane and ethyl acetate fractions from *O. crenata* aerial parts led to the identification of eleven compounds including indole‐3‐carboxylic acid, *n*‐butyl palmitate, tyrosol, L‐rhamnonic acid‐1,4‐lactone, *β*‐sitosterol/stigmasterol mixture, *β*‐sitosterol/stigmasterol glycosides mixture, chrysoeriol, luteolin, apigenin, crenatoside, and verbascoside [59], which were in accordance with the reported compounds in our current study. In a similar study, four alkaloids namely, lupanine, spartein, isosparteine and oxosparteine were defined from *O. aegyptiaca* and *O. ramosa* extracts [78].

### 3.3 Biological Activity

#### 3.3.1 Antioxidant Effects

Recent interest has grown in discovering plant antioxidant compounds as a safer alternative to synthetic ones. Plant bioactive compounds, including phenolics and flavonoids, possess strong antioxidant properties, suggesting that the increased consumption of natural food helps to reduce the risk of chronic disease [79, 81]. In the current study, the *O. racemosa* extracts were assessed for their antioxidant properties using different assays, namely DPPH, ABTS, CUPRAC, FRAP, MCA, and PBD assays. The results are illustrated in Table 3, which shows notable variation among the solvents used and is strongly consistent with the TPC and TFC. The EtOH extract of *O. racemosa* consistently exhibited the most potent antioxidant activity in almost all tested assays, including CUPRAC 1010.44 mg TE/g, ABTS 597.09 mg TE/g, DPPH 514.44 mg TE/g, FRAP 491.20 mg TE/g, and PBD 3.40 mmol TE/g. This strong performance aligned with its previously observed phenolic and flavonoid contents, suggesting they are responsible for the high antioxidant potential [82]. With the same order, EtOH/water showed the second highest antioxidant potential across all assays, followed by water extracts. These results are also consistent with its TPC and TFC levels, supporting the relationship between phenolic and flavonoid contents and antioxidant capacity. In an earlier study, we demonstrated that the extraction solvent significantly affects the antioxidant activity of herbal extracts. Extracts obtained using ethanol and hydroethanol notably showed high levels of TPC and TFC, leading to strong antioxidant potential [83, 85]. In case of MCA, water extracts showed the most vigorous activity, measuring 20.40 mg EDTAE/g, which may be influenced by another phytochemical rather than phenolic and flavonoids, suggesting that not all antioxidant properties solely depend on phenolic and flavonoids. A former study demonstrated that extracts from *Orobanche* species showed positive results in a range of biological activities, including antioxidant, antifungal, antibacterial, anti‐inflammatory, and memory‐enhancing [86]. Renna et al. claimed *O. crenata* Forssk. showed a strong antioxidant property and may be regarded as a highly nutritious food source [87].

**TABLE 3:** Reducing, metal chelating, and total antioxidant (by phosphomolybdenum) effects of the tested extracts.

Extracts	CUPRAC, mg TE/g	FRAP, mg TE/g	MCA, mg EDTAE/g	PBD, mmol TE/g
EA	477.76 ± 13.16^d^	227.00 ± 4.18^d^	10.66 ± 0.24^d^	2.56 ± 0.12^c^
EtOH	1010.44 ± 13.35^a^	491.20 ± 18.93^a^	16.16 ± 0.19^c^	3.40 ± 0.05^a^
EtOH/water	864.88 ± 14.50^b^	438.40 ± 1.93^b^	19.38 ± 0.04^b^	3.05 ± 0.02^b^
Water	529.42 ± 7.01^c^	282.38 ± 2.79^c^	20.40 ± 0.20^a^	2.44 ± 0.01^c^

*Note*: Values are reported as mean ± SD of three parallel measurements.

Abbreviations: EDTAE: EDTA equivalent; TE: trolox equivalents. Different letters indicate significant differences between the tested extracts (*p* < 0.05).

Certain literature records have shown the phytochemical nature and biological activities of the genus *Orobanche* and from which we may discuss the following. DPPH and ABTS assays were used to examine the antioxidant activity of the methanol and aqueous extracts of two parasite plants, *O. crenata* and *Orobanche foetida*, that were extracted from faba bean fields. With IC_50_ values of 2.76 and 7.96 μg/mL, respectively, the *O. crenata* methanol extract demonstrated the highest degree of DPPH and ABTS free radical scavenging capabilities. The total amounts of tannins and polyphenols in the various plant extracts ranged from 0.09–0.32 mg EC/g DW for tannins and from 3.02 to 19.99 mg GAE/g DW for polyphenols. Antimicrobial activity was investigated with the disc diffusion method. The methanol extract of *O. foetida* showed activity against all tested bacterial strains, except *S. aureus* ATCC 6538, by forming clear inhibition zones with diameters between 12 and 30 mm, whereas methanol extracts of *O. crenata* inhibit only *L. monocytogenes* and *S. enteredis* ATCC 502 with an inhibition zone of 10 and 25 mm, respectively [56]. Moreover, *Cistanche violacea*, *O. crenata*, and *O. lavandulacea*, three members of the Orobanchaceae family, were the subjects of the study. DPPH, ABTS, and FRAP assays were used to assess the antioxidant activity of the extracts of hexane, ethyl acetate, acetone, methanol, and water that were produced by sequential maceration. Every extract exhibited antioxidant activity, and the variations among extracts made from various plants and with various solvents have been noted.

#### 3.3.2 Enzyme Inhibitory Effects

Enzymes play a key role in the living body, but their overproduction in the body leads to severe disorders like AChE, BChE, amylase, and glucosidase, which increase in frequency with age, causing Alzheimer's disease and diabetes mellitus [88, 91]. The present study is a preliminary study that shows the enzyme inhibition activity of the *O*. *racemosa* extracts, examined using different enzyme inhibitory assays, namely AChE, BChE, tyrosinase, amylase, and glucosidase. All the studied extracts of *O*. *racemosa* showed variable results depending on the solvents used (Table 4).

**TABLE 4:** Enzyme inhibitory effects of the tested extracts.

Extracts	AChE, mg/GALAE	BChE, mg GALAE/g	Tyrosinase, mg KAE/g	*α*‐Amylase, mmol ACAE/g	*α*‐glucosidase, mmol ACAE/g
EA	2.14 ± 0.24^b^	0.17 ± 0.03^a^	56.01 ± 0.57^b^	0.49 ± 0.01^a^	na
EtOH	2.88 ± 0.05^a^	0.15 ± 0.03^a^	53.09 ± 0.02^c^	0.33 ± 0.01^b^	1.35 ± 0.02^b^
EtOH/water	2.35 ± 0.03^b^	Na	59.66 ± 0.21^a^	0.31 ± 0.01^b^	1.42 ± 0.01^a^
Water	0.04 ± 0.01^c^	na	40.11 ± 0.37^d^	0.09 ± 0.02^c^	0.20 ± 0.01^c^

*Note*: Values are reported as mean ± SD of three parallel measurements.

Abbreviations: ACAE: acarbose equivalent; GALAE: galantamine equivalent; KAE: kojic acid equivalent; and na: not active. Different letters indicate significant differences between the tested extracts (*p* < 0.05).

The EtOH extract recorded the highest AChE inhibition activity, 2.88 mg GALAE/g, followed by EtOH/water extract, 2.35 mg GALAE/g, and EA extracts, which recorded 2.14 mg GALAE/g. The EA showed comparatively the highest BChE inhibition activity, measuring 0.17 mg GALAE/g, while EtOH/water and water extracts showed no inhibition activity. Regarding tyrosinase, EtOH/water extract demonstrated the highest inhibition, measuring 59.66 mg KAE/g, which was closely followed by EA extract, measuring 56.01 mg KAE/g. Consistent with the previous studies, *O. cernua* possesses the main compound acteoside, demonstrating antiphoto‐aging effects [92, 93]. In the case of carbohydrate digestive enzymes, amylase and glucosidase, all the extracts demonstrated less inhibitory activity. Among the studied extracts, EA exhibited the highest antiamylase activity, measuring 0.49 mmol ACAE/g, despite showing no activity against glucosidase. The EtOH/water extract showed the comparatively highest antiglucosidase activity, measuring 1.42 mmol ACAE/g.

#### 3.3.3 Carbonic Anhydrase Inhibition

Carbonic anhydrases (CA) are widespread metalloenzymes responsible for many vital physiological activities [94, 95], and their abnormal activity leads to various disorders. Due to these reasons, CA isoenzymes become targets for drug development, with activators being explored for conditions like Alzheimer's and age‐related disease. However, CA inhibitors have been used to treat conditions like glaucoma, epilepsy, obesity, and cancer [96, 97]. The inhibitory activity of *O. racemosa* extracts against carbonic anhydrase isoform CA I and CA II was evaluated based on their IC50 value. EA extracts showed the highest inhibition of both isoforms, with the lowest IC50 of 16.51 and 16.90 µg/mL for CA I and CA II, respectively (Table 5). Comparatively to Water extracts, the least inhibition was demonstrated, particularly against CA II, measuring IC50 79.65 µg/mL for CA I and 141.42 µg/mL for CA II. *O. racemosa* extracts are rich in bioactive compounds, which may be responsible for inhibiting both isoforms of CA. As previously discussed, phenolic and flavonoids are responsible for the inhibition of human CA [98, 99].

**TABLE 5:** Inhibitory effects of the tested extracts on Human carbonic anhydrase isoenzymes I and II (hCA I and hCA II).

Extracts	CA I		CA II	
	IC_50_, µg/mL	*R* ^2^	IC_50_, µg/mL	*R* ^2^
EA	16.51 ± 0.55^a^	0.9667	16.90 ± 0.91^a^	0.9461
EtOH	64.76 ± 3.43^b^	0.9471	38.07 ± 1.92^b^	0.9575
EtOH/water	67.94 ± 5.36^b^	0.9211	50.58 ± 3.18^c^	0.9372
Water	79.65 ± 5.68^c^	0.9287	141.42 ± 8.82^c^	0.9376

*Note*: Values are reported as mean ± SD of three parallel measurements. Different letters indicate significant differences between the tested extracts (*p* < 0.05).

#### 3.3.4 Multivariate Analysis

**FIGURE 4:**
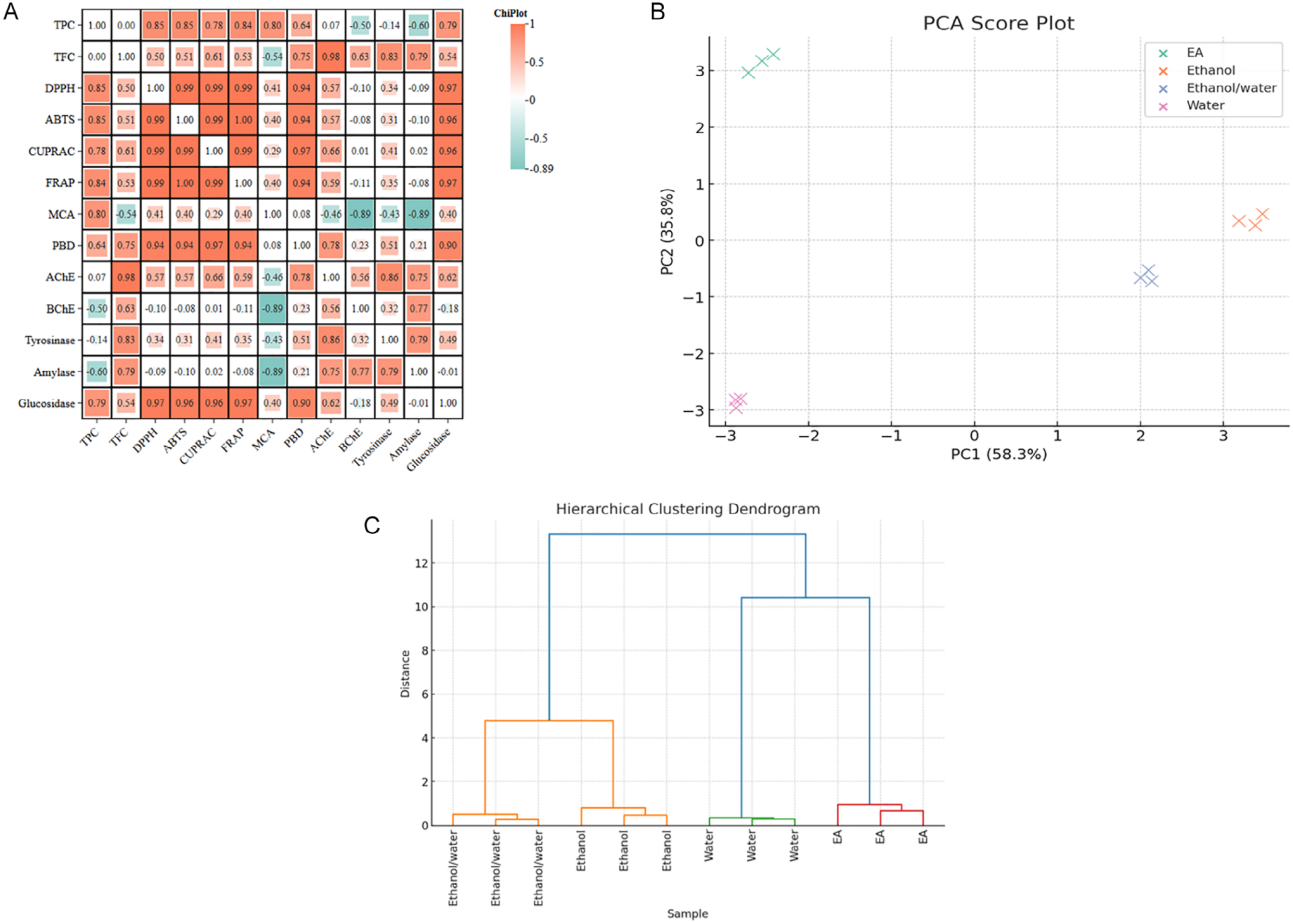
Multivariate analysis based on biological activity results. (A) Pearson correlation analysis; (B) the distribution of extracts in PCA based on biological activity results; and (C) hierarchical cluster analysis.

The multivariate analysis offered additional insights into how the phytochemical and bioactivity metrics of the tested extracts relate to each other (Figure 4). The correlation matrix indicated strong positive relationships among the antioxidant tests (DPPH, ABTS, CUPRAC, and FRAP), implying these assays rely on similar phenolic and flavonoid profiles. Moderate correlations were identified between specific enzyme inhibitory activities, such as amylase and glucosidase, suggesting an overlap of bioactive compounds contributing to antidiabetic potential. Principal component analysis showed that the initial two principal components accounted for a large share of the overall variance, effectively distinguishing the extracts by their chemical profile and bioactivity. Specifically, ethanol‐based extracts were grouped in areas linked with higher phenolic levels and stronger antioxidant actions, while ethyl acetate extracts were aligned with lower bioactivity scores. Hierarchical cluster analysis corroborated this distinction by organizing the samples according to the type of solvent used for extraction, thus highlighting the significant impact of solvent polarity on both chemical composition and corresponding biological effects. This comprehensive statistical approach affirms the interconnectedness of antioxidant and enzyme inhibitory actions with phytochemical abundance, emphasizing the crucial influence of solvent choice on shaping extract bioactivity profiles.

#### 3.3.5 Cell Viability Effect

The MTT assay was used to assess the effects of *O. racemosa* extracts, tested at 100 μg/mL for 72 h, on the viability of two cancer cell lines, HepG2 and SH‐SY5Y, and the nontumoral HEK293 cell line. The results are presented in Table 6.

**TABLE 6:** Cytotoxicity of the samples on HEK293, HepG2, and SH‐SY5Ycell lines.

Extracts	HEK293	**HepG**_**2**_	SH‐SY5Y
Ethyl acetate	9.55 ± 0.87^c^	30.29 ± 2.60^b^	5.53 ± 0.51^c^
Ethanol	12.48 ± 0.65^c^	27.26 ± 2.67^b^	7.45 ± 0.23^c^
Ethanol/water	132.63 ± 3.94^a^	40.56 ± 2.40^a^	69.02 ± 2.11^a^
Water	94.41 ± 3.17^b^	28.86 ± 2.75^b^	50.59 ± 2.98^b^

*Note*: Values are expressed as mean ± SD from three independent experiments, each performed in technical triplicate (*n* = 3 independent experiments). Data were analyzed by two‐way ANOVA followed by Tukey's multiple‐comparisons test (*p* < 0.05).

The EA and EtOH extracts markedly reduced cell viability in all cell lines, indicating strong but nonselective cytotoxic effects. The EA extract resulted in viabilities of 9.55% in HEK293 cells, 30.29% in HepG2 cells, and 5.53% in SH‐SY5Y cells. Similarly, the EtOH extract reduced cell viability to 12.48% in HEK293, 27.26% in HepG2, and 7.45% in SH‐SY5Y cells. Although these extracts showed pronounced cytotoxicity against both cancer cell lines, their strong effects on nontumoral HEK293 cells indicate a lack of tumor selectivity. Therefore, their activity cannot be considered selectively anticancer based on the present results and may instead reflect broad cytotoxic effects on cellular processes shared by both malignant and nonmalignant cells. The present SH‐SY5Y results were obtained at a single concentration of 100 μg/mL, whereas Qu et al. reported an IC_50_ value of 55.89 μg/mL for *O. cernua* essential oil. Since percentage viability at a single concentration and IC_50_ are different endpoints obtained under different experimental conditions, these values cannot be used to compare relative cytotoxic potency. The HEK293 reductions to 9.55% and 12.48% viability motivate the interpretation of EA and EtOH as broadly cytotoxic rather than tumor‐selective.

In contrast, the EtOH/water extract showed the highest viability values in all three cell lines, reaching 132.63% in HEK293, 40.56% in HepG2, and 69.02% in SH‐SY5Y cells. The increase in MTT signal above 100% observed in HEK293 cells may indicate enhanced metabolic activity or a possible proliferative response. However, since the MTT assay primarily reflects mitochondrial metabolic activity rather than directly measuring cell proliferation [100], this apparent proliferative effect should be interpreted with caution and confirmed using complementary approaches that directly assess cell proliferation and long‐term reproductive survival, such as BrdU/EdU incorporation and clonogenic assays [101, 102].

The water extract resulted in viability values of 94.41% in HEK293, 28.86% in HepG2, and 50.59% in SH‐SY5Y cells. Compared with the EA and EtOH extracts, it was markedly less cytotoxic to SH‐SY5Y cells and showed a similar effect on HepG2 cells, while having a much lower effect on nontumoral HEK293 cells. This single‐concentration result suggests different sensitivities among the cell lines but does not establish tumor selectivity. This pattern resembles the differential tumor/nontumour sensitivity described for *O. cernua* essential oil, where SH‐SY5Y cells showed an IC_50_ of 55.89 μg/mL while normal BV‐2 microglial cells were nearly an order of magnitude less sensitive (IC_50_ of 482.1 μg/mL) [103], and aligns with the absence of in vitro cytotoxicity reported in normal human skin fibroblasts exposed to an ethanolic extract of the related *O. minor* [104]. Although the present single‐concentration data cannot yet establish a selectivity index, the magnitude and direction of the difference observed for the water extract are consistent with the pattern reported across other Orobanche taxa tested under comparable MTT protocols. Nevertheless, because the present screening was conducted at a single concentration and exposure time, further dose–response studies are required before conclusions regarding tumor selectivity can be established.

The differences observed among the extracts may be related to their distinct phytochemical compositions and to additive or synergistic interactions among their constituents. Phytochemical surveys across the genus indicate a consistent dominance of lupanine‐type quinolizidine alkaloids and phenylpropanoid glycosides, particularly verbascoside [105], with phenylpropanoid glycosides such as acteoside, 2′‐*O*‐acetylacteoside, verbascoside, and orobanchoside having been isolated from *O. ramosa* (= *Phelipanche ramosa*) parasitizing on tomato [106]. The pronounced nonselective cytotoxicity of the EA and EtOH extracts may therefore reflect an enrichment of mid‐polarity cytotoxic constituents, including alkaloids and less polar phenylpropanoid derivatives, capable of affecting fundamental cellular functions in both cancer and nontumour cells, whereas the comparatively more favourable profile of the water extract is consistent with a partitioning of polar phenylpropanoid glycosides that preferentially affect malignant cells. This interpretation is in agreement with the polarity‐dependent bioactivity pattern reported by Ben Attia et al. [58], who showed that sequential hexane, ethyl acetate, acetone, methanol, and water fractions of *O. crenata* and *O. lavandulacea* produced markedly different cytotoxic profiles despite being derived from the same plant material. Chemotaxonomic context supports this view: phytochemical studies of *O. crenata* itself have identified 22 molecules with predicted antitumour activity [107], and *O. lavandulacea* from Tunisia was reported to be highly cytotoxic on B16F10 melanoma cells, while *O. crenata* was not cytotoxic in the same panel [58]. Such species‐ and compound‐dependent variability across the genus reinforces the view that bioactivity cannot be readily extrapolated across *Orobanche* taxa.

The present MTT data should also be interpreted in the light of methodological variables that are known to influence comparisons across studies. Exposure times in the *Orobanche* cytotoxicity literature range from 24 h, as used for *O. cernua* essential oil on SH‐SY5Y and BV‐2 cells (Qu et al., 2019 [103]) to 48 h protocols [108]. The 72 h exposure used here is at the longer end of the typical range and may therefore accentuate cytotoxicity relative to shorter protocols. In addition, of ≈200 *Orobanche* species recognized worldwide, only 26 have so far been investigated phytochemically and a much smaller subset has been evaluated for cytotoxicity [86]; to our knowledge, no previous study has reported MTT‐derived viability values for *O. racemosa* extracts against HepG2, SH‐SY5Y or HEK293 cells under directly comparable conditions, so the present data represent an original contribution while the comparative interpretation necessarily relies on related *Orobanche* taxa. Taken together with previous reports on other *Orobanche* species, these findings highlight the biological potential of *Orobanche*‐derived extracts and reinforce the importance of evaluating dose dependence, mechanisms of action, and selectivity for cancer cells in future studies.

### 3.4 Computational Study

#### 3.4.1 Molecular Docking

**FIGURE 5:**
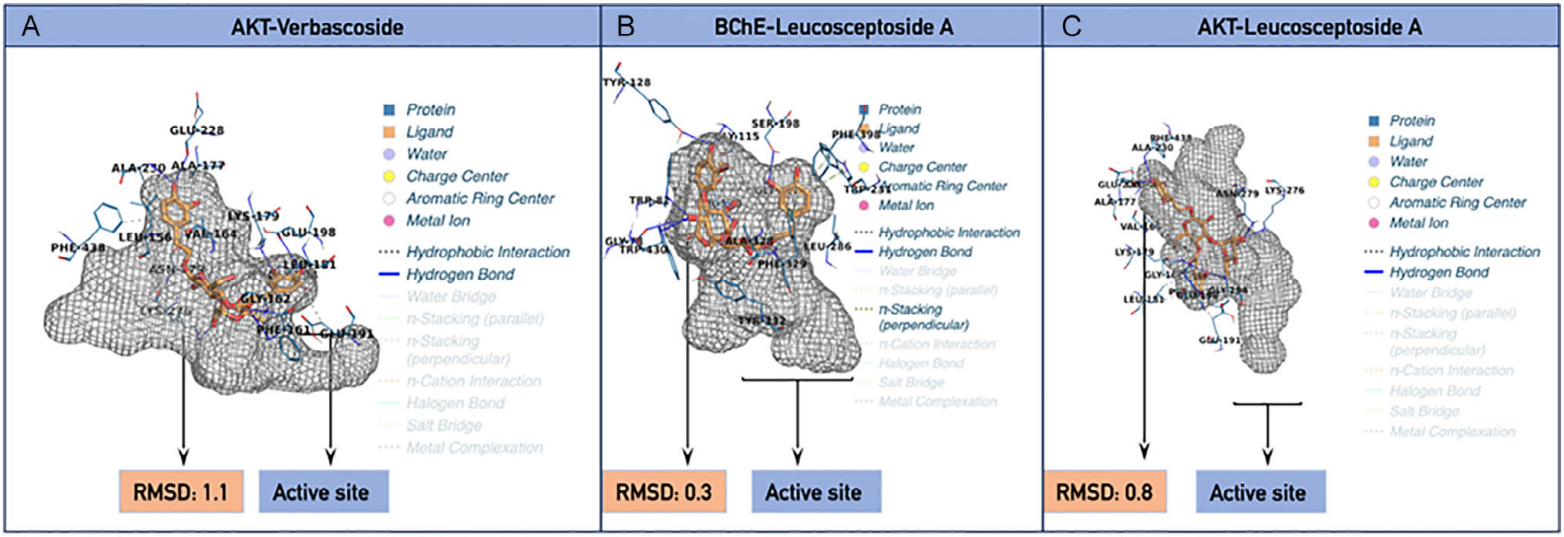
Representative docking poses of top‐scoring ligand–target complexes within their active sites. (A) AKT‐Verbascoside. (B) BChE‐Leucosceptoside A. (C) AKT‐Leucosceptoside A.

A total of 384 compound–target combinations (16 phytochemicals × 24 targets) were evaluated and presented in Figure S1. Of these, 251 unique complexes with docking scores of −7.0 kcal/mol or lower were retained for detailed interaction analysis and reported in Table S2. Complexes with scores above −7.0 kcal/mol were included in the heatmap but not in Table S2. Examination of the binding energies revealed that 251 complexes achieved values of −7 kcal/mol or lower; this finding indicates that a substantial proportion of the compounds possess high‐affinity potential for the target proteins. Pose quality was also supported by RMSD metrics. RMSD analyses demonstrated the redocking sensitivity of the complexes and the stability of their binding conformations. RMSD values ranged from 0.01 to 57.4 Å; systems with low RMSD (1–2 Å) stood out with high conformational agreement and reliable binding poses. Notably, the generally low RMSD values of the top five complexes with the highest affinity support the reliability of the docking scores. The highest binding score was observed for the Verbascoside–AKT complex at −10.7 kcal/mol, and this pose aligns well with an RMSD of 1.07 Å (Figure 5A). This was followed by the Leucosceptoside A‐BChE (−10.5 kcal/mol; RMSD 0.28 Å) (Figure 5B), Verbascoside‐BChE (−10.3 kcal/mol; RMSD 0.62 Å), Leucosceptoside A‐AKT (−10.3 kcal/mol; RMSD 0.83 Å) (Figure 5C), and Orobanchoside‐Keap1 (−10.2 kcal/mol; RMSD 0.56 Å) complexes (Figure S1 and Table S2).

Among the retained complexes, 10 involved AChE, with the most frequent interactions observed at residues TYR124, TYR133, TRP86, HIS447, and SER20. The Epicatechin–AChE complex (7E3H, −9.7 kcal/mol; RMSD 1.01 Å) engaged critical residues near the active site, presenting a binding model that could potentially inhibit acetylcholine hydrolysis. For BChE, all 16 compound–target combinations met the docking‐score threshold and were retained for detailed analysis, with TRP82, GLY117, THR120, GLY115, and TYR332 identified as key interacting residues. Both Leukosceptoside A and Verbascoside formed an extensive network of hydrogen bonds and hydrophobic interactions within the catalytic gorge, a profile consistent with the strong AChE inhibition observed for the EtOH extract and the potent BChE inhibition shown by the EA extract, as reported in the literature to be linked to cognitive function support [109]. For α‐amylase, complexes were evaluated, with TRP59, GLU233, TYR62, GLN63, and HIS305 emerging as the key interacting residues. For α‐glucosidase, complexes were analyzed, with ARG352, GLY253, LYS283, TYR291, and SER290 identified as the most frequent binding residues. In both enzymes, Eriodictyol‐hexoside stood out as the lead compound (α‐amylase 2QV4: −9.4 kcal/mol; RMSD 1.03 Å; α‐glucosidase 7KBJ: −9.8 kcal/mol; RMSD 1.12 Å). The ligand formed multiple hydrogen bonds and hydrophobic interactions with catalytically relevant residues; TRP59, GLU233, and TYR62 in α‐amylase, and TYR291, ARG352, and GLY253 in α‐glucosidase. For hCA I, complexes highlighted HIS94, HIS96, HIS119, THR199, and VAL143, while for hCA II, complexes revealed HIS94, HIS96, HIS119, THR199, and VAL121 as the primary interacting residues. Leukosceptoside B showed strong occupancy of the Zn^2+^ binding pocket in both isoenzymes (−9.3 to −9.0 kcal/mol), providing a mechanistic basis for isoenzyme‐selective CA inhibition [110]. For Keap1, complexes were evaluated, with VAL465, THR560, ILE559, VAL606, and VAL561 emerging as frequent contact points. The Orobanchoside‐Keap1 complex exhibited an extensive hydrogen bond and hydrophobic interaction network, suggesting—albeit based solely on in silico evidence—a potential binding mode that might facilitate NRF2 release and the activation of antioxidant gene programs [111]. For AKT, 16 complexes were analyzed, with ALA230, VAL164, GLY162, LYS179, and PHE161 identified as frequent interaction residues. Verbascoside achieved the highest docking score (−10.7 kcal/mol; RMSD 1.07 Å). For CDK2 (GLU81, LEU83, ASP145, PHE82, LYS89) and CDK4 (VAL96, ALA33, LYS35, ASP97, and GLN98), Eriodictyol‐hexoside was predicted—based solely on in silico evidence—to bind with high affinity within the ATP‐binding clefts [112, 113]. For p38 MAPK (8 complexes; LYS53, MET109, ASN155, ALA157, and PHE169) and Cyclin D1 (GLN253, ASN254, LEU255, GLU256, and THR262), the predicted binding modes involved multiple hydrogen bonds and hydrophobic interactions with key residues, suggesting a potentially strong binding profile. In PD‐L1 (15 complexes; LYS78, ASP77, ARG86, TYR68, and ASN66) and NFκB (LYS221, GLU222, ARG246, TYR254, and GLN247), these poses represent predicted binding configurations and do not demonstrate modulation of immune‐checkpoint or inflammatory signalling. For BCL‐2 (ARG146, GLU136, ASP111, ASN143, and PHE104) and Caspase‐3 (CYS163, HIS121, GLU123, ARG207, SER205), the predicted interactions were consistent with key catalytic or regulatory regions. In Caspase‐9, the two evaluated complexes—Eriodictyol hexoside (−7.4 kcal/mol; 1.05 Å) and Leucosceptoside B (−7.2 kcal/mol; 1.18 Å)—were predicted to engage primarily GLU143, SER144, ARG146, GLY147, and the C‐terminal PHE399‐THR402‐LYS401 region through hydrogen bonding and hydrophobic contacts. By contrast, the poses for TERT with 8‐O‐methylretusin (−8.8 kcal/mol; RMSD 8.52 Å) and BRPF1 with Leucosceptoside A (−7.5 kcal/mol; RMSD 5.83 Å) displayed high RMSD values, limiting their reliability. Nras (−7.6 kcal/mol; RMSD 1.56 Å) and PPARγ (−7.2 kcal/mol; RMSD not determined) represent plausible binding hypotheses, but these remain purely computational predictions requiring experimental validation.

Collectively, the docking analysis generated a set of multitarget interaction hypotheses involving enzymes and signalling proteins. Favourable docking scores and predicted contacts can support prioritisation for follow‐up work, but they do not establish protein inhibition, pathway modulation, cytotoxic selectivity, or therapeutic activity. Experimental validation using purified‐protein assays, cellular target‐engagement or pathway measurements, and dose–response testing is required before mechanistic or pharmacological conclusions can be drawn.

#### 3.4.2 MD Simulations

**FIGURE 6:**
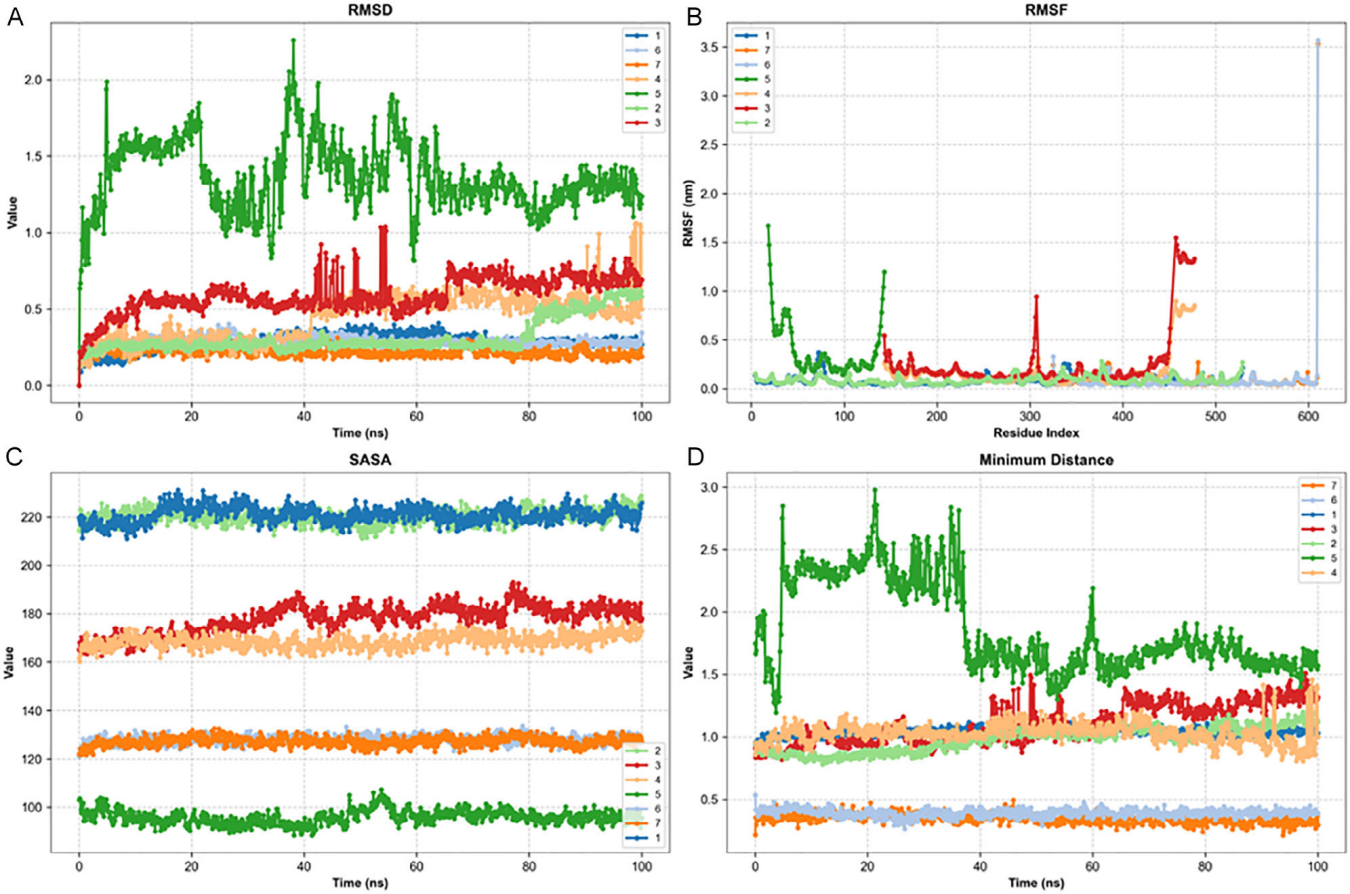
Representation of molecular dynamics simulations in graphical form; (A) RMSD. (B) RMSF. (C) SASA. (D) Minimum distance.

**FIGURE 7:**
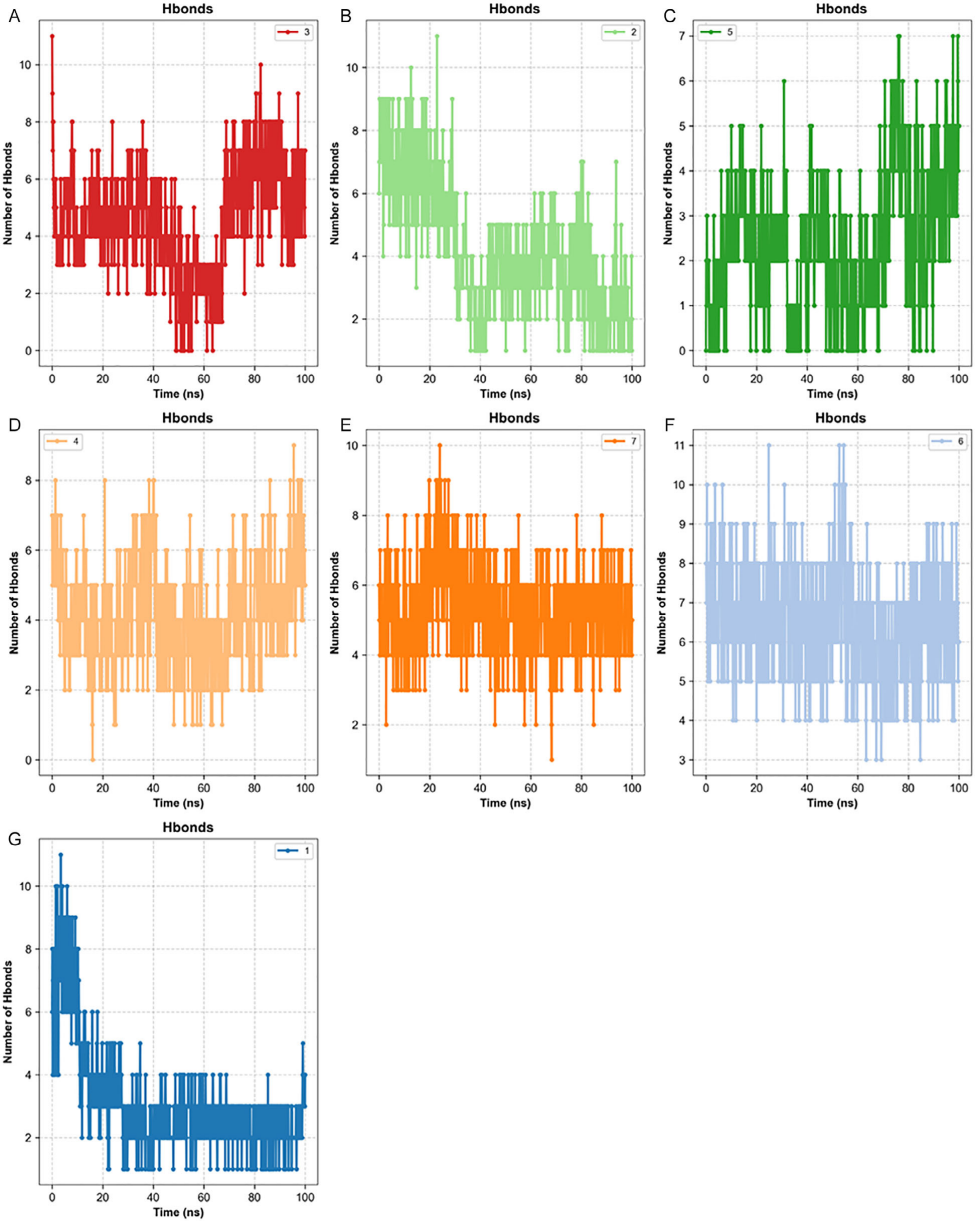
Time‐dependent hydrogen bond analysis for each protein–ligand complex.

MD simulations are widely used as a powerful computational technique to evaluate the structural stability of protein–ligand complexes and the temporal behaviour of their binding interactions [114]. In this study, 384 theoretical compound–target combinations were screened, and 251 unique complexes with docking scores of −7.0 kcal/mol or lower were retained after the duplicated entries were removed. Seven representative high‐scoring systems were selected by considering docking score, target coverage, and biological relevance; these systems did not represent the seven strongest complexes overall. The selected systems were BChE‐Leucosceptoside A (C1), BChE‐Verbascoside (C2), AKT‐Leucosceptoside A (C3), AKT‐Verbascoside (C4), PD‐L1‐Verbascoside (C5), Keap1‐Verbascoside (C6), and Keap1‐Eriodictyol‐hexoside (C7). The selected complexes were subjected to 100 ns MD simulations, and parameters including RMSD, root mean square fluctuation (RMSF), solvent‐accessible surface area (SASA), minimum binding distance, and hydrogen bonds (H‐bonds) were comparatively analyzed. The results were evaluated based on binding distance magnitude and stability, average number of hydrogen bonds, and RMSF profiles to determine the stability levels of the complexes (Figures 6 and 7).

Among the analyzed trajectories, C7 showed an average minimum ligand–protein distance of 0.35 nm, five hydrogen bonds, and RMSF values generally below 0.12 nm. The protein RMSD averaged 0.22 nm, while SASA remained around 127.2 nm^2^. Under the simulated conditions, these values indicated comparatively limited structural deviation, without establishing experimental binding stability or biological activity. The most mobile regions in this complex were residues 610, 481, 384, 385, and 325. C6 showed an average minimum ligand–protein distance of 0.39 nm, approximately seven hydrogen bonds, and consistently low residue fluctuations throughout the simulation. The protein RMSD averaged 0.29 nm, while SASA averaged 127.9 nm^2^, indicating comparatively limited structural deviation under the simulated conditions. The most mobile residues were 610, 325, 385, 384, and 348. C2 showed an average minimum ligand–protein distance of 0.98 nm and approximately four hydrogen bonds. Its RMSF values were generally around 0.15 nm, the protein RMSD averaged 0.32 nm, and SASA averaged 220.0 nm^2^. These results describe the relative structural behaviour of C2 only under the simulated conditions. The most mobile regions were residues 377, 529, 378, 379, and 380. C3 had an average minimum of 1.10 nm and formed about five hydrogen bonds. The average RMSF and RMSD values were 0.16 and 0.59 nm, respectively, while the mean SASA was 177.7 nm^2^. The most mobile regions were residues 457, 458, 459, 465, and 466. C4 had an average minimum ligand–protein distance of 1.03 nm and formed about four hydrogen bonds. The average RMSF and RMSD values were 0.15 and 0.44 nm, respectively, while the mean SASA was 168.7 nm^2^. The number of hydrogen bonds fluctuated during the simulation. The most mobile residues were 457, 477, 465, 464, and 466. C1 had an average minimum ligand–protein distance of 1.04 nm and formed about three hydrogen bonds. The average RMSF and RMSD values were 0.17 and 0.28 nm, respectively, while the mean SASA was 220.9 nm^2^. The number of hydrogen bonds decreased during the later stages of the simulation. The most mobile residues were 72, 73, 74, 71, and 75. C5 had an average minimum ligand–protein distance of 1.86 nm and formed about two hydrogen bonds. The average RMSF and RMSD values were 0.19 and 1.36 nm, respectively, while the mean SASA was 95.9 nm^2^ and varied during the trajectory. C5 showed greater structural deviation than the other analyzed systems. The most mobile regions were residues 18, 19, 20, 143, and 21. Additionally, its high SASA variability indicated instability of the binding surface. The hydrogen bond profile of C2, although initially high, showed a marked decrease over time, with RMSF values rising to ≈1.8 Å. Taken together, the MD simulations revealed different degrees of structural persistence among the seven representative complexes under the simulated conditions. C7 and C6 showed comparatively lower protein RMSD values and shorter minimum ligand–protein distances, whereas C5 displayed greater deviation during the trajectory [115] (Figures 6 and 7). These observations are limited to the analyzed simulations and should not be interpreted as evidence of experimental binding affinity, target inhibition, pathway modulation, cellular efficacy, or therapeutic activity. Replicate simulations and experimental validation are required to test these computational predictions.

#### 3.4.3 Limitations of the Computational Analyses

The docking and MD analyses are predictive and should be interpreted as hypothesis‐generating. AutoDock Vina scores provide relative ranking within the selected protocol but do not constitute experimental dissociation constants or proof of target inhibition. Pose RMSD is a geometric measure and does not establish biological validity. Moreover, each MD system was represented by a single 100 ns trajectory without replicate simulations or an endpoint free‐energy calculation; apparent structural persistence therefore cannot demonstrate biochemical activity, cellular pathway modulation, selectivity, bioavailability, toxicity, or therapeutic efficacy. The prioritized complexes should consequently be tested using purified‐protein or enzyme‐inhibition assays, orthogonal target‐engagement methods, dose–response and selectivity studies, and relevant cellular pathway analyses before mechanistic or pharmacological conclusions are made.

## 4 Conclusion

The current study is the first to examine the chemical profile and biological activity of *O*. *racemosa* EA, EtOH, EtOH/water, and water extracts. This study highlights the potential value of scientificallyexploring *O*. *racemosa*, which leads to promising results. Among the studied extracts, the EA extract contained the highest number of compounds (20), followed by the water extract (19), as determined by HPLC–MS/MS. All the extracts, especially EtOH and EtOH/water extracts, exhibited the highest phenolic and flavonoid contents. These extracts also demonstrated the most potent antioxidant ability across different assays, which may be attributed to their highest TPC and TFC. Furthermore, this is the first study to report *O*. *racemosa* extracts’ inhibitory activity against enzymes and CA associated with neurodegenerative diseases, skin‐related disorders, diabetes, and obesity. In the cell viability assay, the EA and EtOH extracts of O. racemosa markedly reduced the viability of both cancer cell lines (HepG2 and SH‐SY5Y) and nontumoral HEK293 cells, indicating strong but nonselective cytotoxicity. Given the absence of dose–response, IC50, and mechanistic data, these findings should be regarded as preliminary and do not support conclusions regarding selective anticancer efficacy. In addition to the experimental findings, molecular docking was used to screen 384 theoretical compound–target combinations. After application of the docking‐score threshold and removal of six duplicated compound–target entries, 251 unique complexes with docking scores of −7.0 kcal/mol or lower were retained. Seven representative high‐scoring complexes (C1–C7), selected by considering docking score, target coverage, and biological relevance rather than representing the seven strongest complexes overall, were subjected to single 100 ns MD simulations. Their trajectory behaviour provides computational hypotheses about relative structural persistence under the simulated conditions and does not establish experimental binding affinity, target inhibition, pathway modulation, selectivity, or therapeutic efficacy. Replicate simulations and biochemical and cellular validation are therefore required. Overall, the current study's findings offer valuable data to support the extraction of *O*. *racemosa*. Further investigation is necessary to characterize the phytochemicals contributing to the biological effect of *O*. *racemosa*.

## Author Contributions

**Nilofar Nilofar**, **Enver Saka**, **Mehmet Veysi Cetiz**, **Gokhan Zengin**: conceptualization. **Enver Saka**, **Mehmet Veysi Cetiz**, **Maria J. Rodrigues**, **Eliana Fernandes**, **Luisa Custodio**, **Ismail Yapıcı**, **Esraa A. Elhawary**, **Omayma A. Eldahshan**, **Gokhan Zengin**: methodology. **Mehmet Veysi Cetiz**: software. **Evren Yildiztugay**, **Abdel Nasser B. Singab**, **Carlos L. Cespedes-Acuna**, **Gokhan Zengin**: validation. **Gokhan Zengin**: formal analysis. **Nilofar Nilofar**, **Enver Saka**, **Mehmet Veysi Cetiz**, **Ilhami Gulcin**, **Gokhan Zengin**: investigation. **Evren Yildiztugay**: resources. **Enver Saka**, **Mehmet Veysi Cetiz**, **Gokhan Zengin**: data curation. **Nilofar Nilofar**, **Eliana Fernandes**, **Luisa Custodio**, **Gokhan Zengin**: writing – original draft preparation. **Maria J. Rodrigues**, **Luisa Custodio**, **Ilhami Gulcin**, **Gokhan Zengin**: writing – review and editing. **Mehmet Veysi Cetiz**: visualization. **Gokhan Zengin**, **Carlos L. Cespedes-Acuna**: supervision. **Gokhan Zengin**: project administration. **Gokhan Zengin**: funding acquisition. All authors have read and agreed to the published version of the manuscript.

## Funding

This research received no external funding. The numerical calculations reported in this paper were fully/partially performed at TUBITAK ULAKBIM, High Performance and Grid Computing Center (TRUBA resources).

## Conflicts of Interest

The authors declare no conflicts of interest.

## Supporting information

Supplementary Material

## Data Availability

Data will be made available on request.
