# Supplementary material for: Comprehensive Chemical and Biological Assessment of Orobanche racemosa Extracts Using Chromatographic, In Vitro, In Silico, and Cell‐Based Techniques
Source: ChemistryOpen. 2026 Aug 17;15(9):e70283. doi: 10.1002/open.70283 (PMC13482041; doi:10.1002/open.70283)
Supplement: Supplementary file 1 [file OPEN-15-e70283-s001.pdf]

UPLC/MS/MS analysis

The phytochemical analysis of *O. racemosa* extracts was assessed according to the previously reported method using high-performance liquid chromatographic (HPLC) analysis joined with an ESI-MS/MS spectrometer detector [34]. This technique allowed tentative identification of phytoconstituents based on the molecular weights. The plant extract (100 µg/ml) was dissolved in methanol (HPLC-grade), then filtered via a membrane disc (0.20 µm). Then, the filtrate (10 µL) was injected into HPLC-ESI-MS/MS. The used HPLC instrument has the following specifications: Waters® stocked with a reversed-phase C-18 column (ACQUITY UPLC-BEH C-18, particle size~1.7µm, dimensions=2.1 × 50 mm). Before injection, the mobile phase was filtered through a membrane disc filter (0.2 µm) and sonicated. The elution run took 35 min using gradient elution (water and methanol acidified with 0.1% formic acid) with a flow rate of 0.2 mL/min. On an XEVO TQD triple quadrupole instrument, positive and negative ions were acquired using ESI-MS. Waters® Corporation, Milford, MA 01757, U.S.A. supplied the HPLC unit and mass spectrometer. Edwards®, U.S.A., provided the vacuum pump at desolvation temperatures of 150 and 440 °C. The mass spectra were obtained using the software Masslynx 4.1 at an ESI range m/z of 100-1000. To tentatively identify the obtained mass spectra, the peak retention time (tR) and their fragmentation pattern were compared with the reported data in the literature.

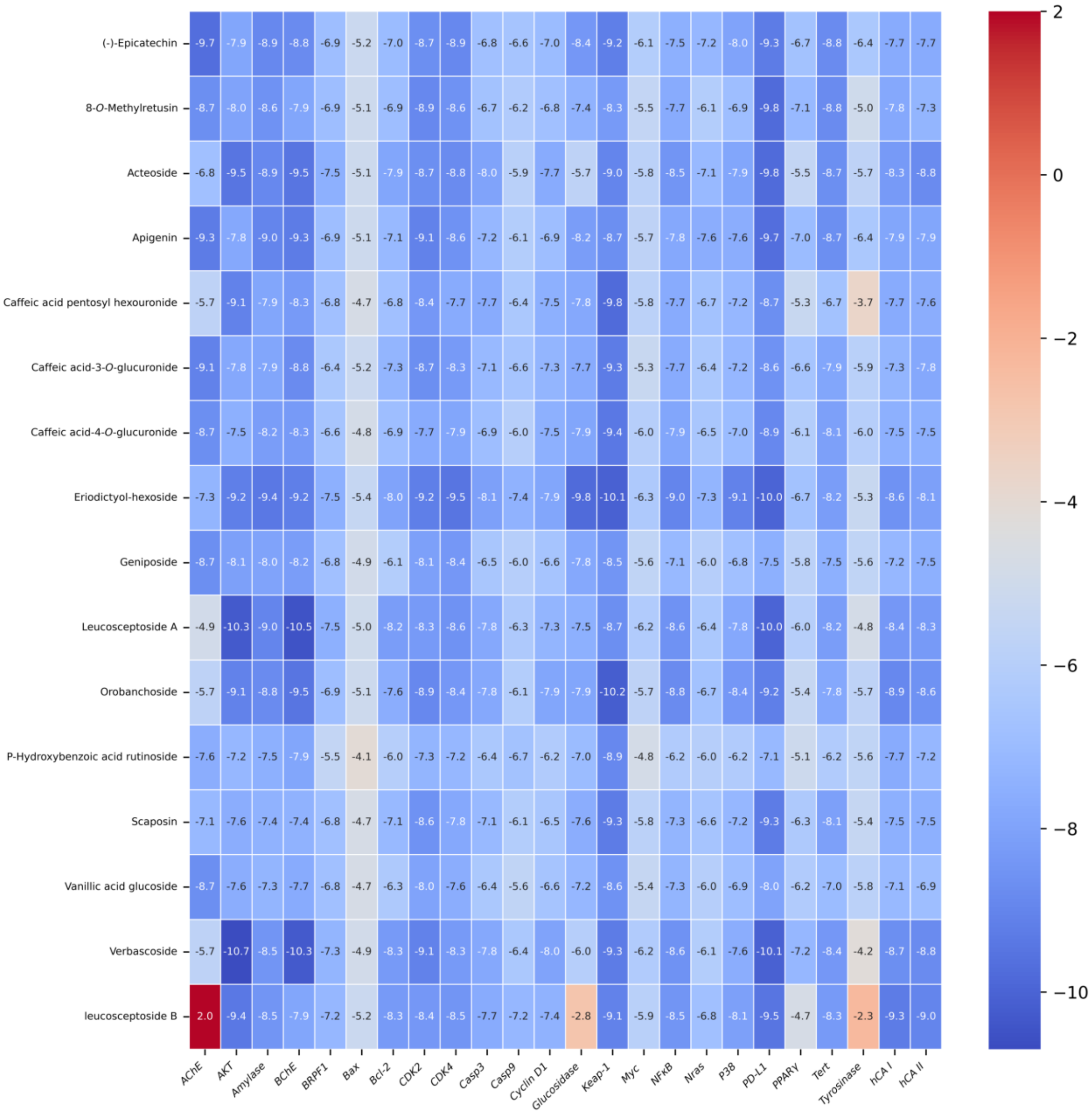

Figure S1: Binding affinity heatmap (kcal/mol) of 16 phytochemicals docked against 24 therapeutic targets. Color scale represents docking scores, with darker blue indicating stronger binding.

Table S1: Docking grid center coordinates and box dimensions for the selected protein targets.

Target Protein	**PDB ID**	**Center X, Y, Z**	**Grid Size X, Y, Z**
AChE	7E3H	-54.44, 32.90, -28.65	25, 25, 25
BChE	6EQP	32.16, -16.33, 40.73	25, 25, 25
Tyrosinase	6QXD	21.81, 12.22, 91.40	25, 25, 25
Amylase	2QV4	14.188, 48.964, 22.886	28, 28, 24
Glucosidase	7KBJ	-18.79, 2.18, 15.75	25, 25, 25
hCA I	3LXE	-19.049, 36.885, 44.812	40, 40, 40
hCA II	4IWZ	14.059, 4.757, 14.338	40, 40, 40
AKT	4GV1	-17.903, 0.789, 15.945	20,38,34
BRPF1	5MWZ	-9.411, 9.221, 6.159	40,40,40
CDK2	6GUE	-6.463, -24.101, 25.309	40,40,40

CDK4	2W96	5.126, -5.071, 74.401	42,64,74
Cyclin D1	2W99	15.991, 26.615, 49.003	26,30,26
Myc	1NKP	71.779, 68.731, 41.5	60,72,40
PD-L1	5N2F	32.621, 17.007, 130.416	30,30,30
TERT	5CQG	3.629, -3.75, -45.599	126,98,126
PPARγ	1I7I	13.814,18.569,16.359	66,46,106
Bax	3PL7	26.657, 17.779, 12.31	40, 112, 40
Bcl-2	6O0K	-13.787, 2.227, -11.244	28, 24, 54
Casp3	3GJQ	28.642, 34.169, 12.047	20, 20, 20
Casp9	1JXQ	11.643, 37.359, 18.522	40, 40, 40
Keap-1	6T7V	-8.29, 21.307, 12.442	60, 74, 40
Nras	8VM2	0.675, -6.511, -9.185	40, 40, 40
P38	1A9U	1.766, 19.665, 32.654	40, 52, 40
NFκB	*4FA6*	42.61, 16.62, 32.82	30, 28, 28

Table S2: The docking score (kcal/mol) and interacting residues of the protein.

Compound	Target	PDB ID	Binding energy	RMSD	Type and Binding site
Verbascoside	AKT	4GV1	-10.7	1.1	Hbond: PHE:161;GLY:162;LYS:179;GLU:198;GLU:228;ALA:230;LYS:276;ASN:279;GLY:294<br>Hydrophobic: LEU:156;PHE:161;VAL:164;VAL:164;ALA:177;LEU:181;LEU:181;GLU:191;PHE:438
Verbascoside	Amylase	2QV4	-8.5	0.5	Hbond: TRP:59;GLN:63;ASP:197;ALA:198;LYS:200;ILE:235;HIS:305<br>Hydrophobic: TRP:59;TRP:59;TRP:59;LEU:162;LYS:200;ILE:235<br>Pi-stacking: HIS:201<br>Salt bridge: HIS:305
Verbascoside	BChE	6EQP	-10.3	0.6	Hbond: TRP:82;GLY:115;GLY:117;THR:120;THR:120;TYR:128;SER:198;TRP:430<br>Hydrophobic: TRP:82;LEU:286;ALA:328;TYR:332;TRP:430<br>Pi-stacking: TRP:82;TRP:82;TRP:231;TRP:231;PHE:329
Verbascoside	BCL2	6O0K	-8.3	1.0	Hbond: GLU:136;GLU:136;ASP:140;ARG:146;ARG:146<br>Hydrophobic: PHE:104;ASP:111;ALA:149;PHE:153<br>Pi-stacking: PHE:104;PHE:104<br>Salt bridge: ARG:146
Verbascoside	BRPF1	5MWZ	-7.3	0.7	Hbond: ASN:651;SER:654;SER:654;GLU:655;GLU:655;GLU:655;TYR:665;ASN:708;ASN:708<br>Hydrophobic: PHE:653;VAL:657;PRO:658;PHE:714;PHE:714
Verbascoside	Casp-3	3GJQ	-7.8	1.0	Hbond: THR:62;SER:63;ARG:207;SER:209;SER:209;PHE:250;SER:251<br>Hydrophobic: TRP:206;TRP:206;PHE:256;PHE:256<br>Pi-stacking: PHE:256<br>Salt bridge: ARG:207;ARG:207
Verbascoside	CDK2	6GUE	-9.1	5.8	Hbond: TYR:180;GLU:12;GLU:12;TYR:15;TYR:15;ASP:86;ASN:132;ASP:145<br>Hydrophobic: VAL:163;ILE:10;TYR:15;VAL:18;LYS:89;LEU:134;ALA:144<br>Salt bridge: ARG:50;ARG:126;ARG:150;LYS:89
Verbascoside	CDK4	2W96	-8.3	6.6	Hbond: ILE:12;VAL:14;ALA:16;LYS:35;ARG:101;ARG:101<br>Hydrophobic: ILE:12;ALA:33;LYS:142;LEU:147;THR:177<br>Salt bridge: ARG:101;LYS:142
Verbascoside	Cyclin D1	2W99	-8.0	0.7	Hbond: CYS:73;CYS:73;CYS:73;GLU:74;GLU:75;GLU:75;PHE:78;GLU:115;GLN:176;GLN:176;GLN:183<br>Hydrophobic: LEU:65;GLU:69;PHE:78<br>Salt bridge: LYS:180
Verbascoside	hCA I	3LXE	-8.7	1.0	Hbond: SER:65;GLN:92;GLN:92;GLN:92;HIS:94;THR:199;HIS:200<br>Hydrophobic: TRP:5;LEU:131;ALA:135;LEU:198;HIS:200;TYR:204<br>Pi-stacking: HIS:94<br>Salt bridge: HIS:94
Verbascoside	hCA II	4IWZ	-8.8	1.6	Hbond: ASN:67;PHE:70;PHE:70;ASP:72;GLN:92;HIS:119;THR:199;THR:199;THR:200;THR:200;THR:200<br>Hydrophobic: GLU:69;VAL:121;VAL:143;LEU:198<br>Pi-stacking: HIS:94
Verbascoside	Keap-1	6T7V	-9.3	2.7	Hbond: VAL:369;VAL:465;ILE:559;THR:560;VAL:561;VAL:606;VAL:606<br>Hydrophobic: VAL:369;VAL:514;VAL:608
Verbascoside	NFκB	4FA6	-8.6	0.6	Hbond: ALA:805;SER:806;LYS:807;LYS:833;THR:887;ASP:950;ASN:951;ASP:964;ASP:964<br>Hydrophobic: ILE:831;TYR:867;ILE:879;ILE:963;ILE:963;ASP:964<br>Salt bridge: LYS:890

Verbascoside	P38	1A9U	-7.6	19.1	Hbond: LYS:66;ARG:70;ARG:70;ILE:147;ARG:149;ALA:172;ALA:172;ALA:172;ARG:173;HIS:174;HIS:174;THR:175;THR:175;TYR:200;TYR:200;GLN:325;GLU:328<br>Hydrophobic: ILE:63;LEU:171;HIS:174;GLU:328<br>Salt bridge: ARG:70;HIS:174
Verbascoside	PD-L1	5N2F	-10.1	0.5	Hbond: ILE:54;ASN:63;GLN:66;VAL:68;GLU:72;ILE:116;SER:117;ALA:121;ALA:121;ASP:122;TYR:123;LYS:124<br>Hydrophobic: ILE:54;TYR:56;TYR:56;VAL:68;MET:115;ALA:121;TYR:123
Verbascoside	PPARγ	1I7I	-7.2	nd	Hbond: LYS:263;LYS:263;GLY:284;ARG:288;SER:342;GLU:343<br>Hydrophobic: ILE:249;PHE:287;PHE:287;ARG:288;LEU:330;LEU:333;ILE:341;ILE:341<br>Pi-stacking: PHE:287
Verbascoside	Tert	5CQG	-8.4	1.0	Hbond: ASP:191;ASN:192;ASN:192;PHE:193;ALA:195;ALA:195;ASP:251;ASP:251;LYS:372<br>Hydrophobic: PHE:193;TRP:302;ASP:343
Vanillic acid glucoside	AChE	7E3H	-8.7	0.9	Hbond: GLY:120;GLY:120;GLY:120;TYR:124;TYR:133;HIS:447<br>Hydrophobic: PHE:297;PHE:338
Vanillic acid glucoside	AKT	4GV1	-7.6	0.2	Hbond: LYS:158;LYS:158;ALA:230;THR:291;ASP:292<br>Hydrophobic: LEU:156;VAL:164;ALA:177;PHE:438
Vanillic acid glucoside	Amylase	2QV4	-7.3	0.8	Hbond: ALA:198;GLU:233;GLU:233<br>Hydrophobic: TRP:58;TRP:59
Vanillic acid glucoside	BChE	6EQP	-7.7	1.1	Hbond: TRP:82;THR:120;TYR:128;TRP:430<br>Hydrophobic: TRP:82;TRP:82
Vanillic acid glucoside	CDK2	6GUE	-8.0	0.8	Hbond: TYR:180;GLU:12;TYR:15;TYR:15;LEU:83;LEU:83;ASP:145;ASP:145<br>Hydrophobic: VAL:163;ILE:10;VAL:18;ALA:31;LEU:134<br>Salt bridge: ARG:50;ARG:126;ARG:150
Vanillic acid glucoside	CDK4	2W96	-7.6	1.0	Hbond: GLY:15;ALA:16;HIS:95;VAL:96;VAL:96;ASP:99;ASP:99;ASP:99;ASP:99;ARG:101;ASP:158<br>Hydrophobic: GLU:144<br>Salt bridge: LYS:142<br>Pi-cation: LYS:35
Vanillic acid glucoside	Glucosidase	7KBJ	-7.2	1.1	Hbond: ASP:251;GLY:253;GLY:254;GLY:266;LYS:283;GLU:287;SER:290<br>Hydrophobic: TYR:291<br>Salt bridge: ARG:334;ARG:352
Vanillic acid glucoside	hCA I	3LXE	-7.1	1.0	Hbond: HIS:64;HIS:119;THR:199;THR:199<br>Salt bridge: HIS:64;HIS:67;HIS:94
Vanillic acid glucoside	Keap-1	6T7V	-8.6	1.0	Hbond: ARG:415;GLY:462;VAL:463;VAL:463;GLY:603;VAL:604;VAL:606
Vanillic acid glucoside	NFκB	4FA6	-7.3	0.1	Hbond: THR:887;LYS:890;ASP:950;ASP:964<br>Hydrophobic: LYS:833;ILE:879;ILE:879;ILE:879;ILE:963<br>Salt bridge: LYS:833
Vanillic acid glucoside	PD-L1	5N2F	-8.0	7.6	Hbond: SER:117;ALA:121<br>Hydrophobic: TYR:56;ALA:121;TYR:123
Vanillic acid glucoside	Tert	5CQG	-7.0	4.6	Hbond: TRP:498;ARG:557;ARG:557<br>Hydrophobic: PHE:494;PHE:494;ILE:497;TYR:551;LEU:554
Scaposin	AChE	7E3H	-7.1	0.7	Hbond: TRP:86;TYR:124;TYR:133<br>Hydrophobic: PHE:338<br>Pi-stacking: TYR:337
Scaposin	AKT	4GV1	-7.6	0.4	Hbond: ALA:230<br>Hydrophobic: VAL:164;VAL:164
Scaposin	Amylase	2QV4	-7.4	1.3	Hbond: GLN:63;HIS:101;HIS:305;HIS:305<br>Hydrophobic: LEU:165;LEU:165

Scaposin	BChE	6EQP	-7.4	0.4	Hbond: TRP:82;GLY:117;SER:198;PRO:285;TRP:430<br>Hydrophobic: TYR:332<br>Pi-stacking: PHE:329
Scaposin	BCL2	6O0K	-7.1	0.7	Hbond: GLU:136;ARG:146;ARG:146;PHE:153<br>Hydrophobic: PHE:104;LEU:137;ALA:149
Scaposin	Casp-3	3GJQ	-7.1	nd	Hbond: HIS:121;GLY:122;SER:205;ARG:207;ARG:207;ARG:207<br>Hydrophobic: TYR:204
Scaposin	CDK2	6GUE	-8.6	0.1	Hbond: TYR:180;LYS:33;LEU:83;ASP:86;LYS:89<br>Hydrophobic: VAL:163;ILE:10;ILE:10;LEU:134<br>Salt bridge: ARG:50;ARG:126;ARG:150
Scaposin	CDK4	2W96	-7.8	0.2	Hbond: ALA:16;LYS:35;ARG:101;ARG:101;LYS:142;THR:177<br>Hydrophobic: GLU:144
Scaposin	Glucosidase	7KBJ	-7.6	4.4	Hbond: LEU:252;GLY:253;ARG:334;TYR:348;PHE:350<br>Hydrophobic: PHE:350<br>Pi-cation: ARG:352
Scaposin	hCA I	3LXE	-7.5	1.1	Hbond: HIS:64;GLN:92;HIS:94;HIS:96;THR:199;HIS:200<br>Hydrophobic: LEU:198;LEU:198;HIS:200<br>Pi-stacking: HIS:94<br>Pi-cation: HIS:200
Scaposin	hCA II	4IWZ	-7.5	1.0	Hbond: ASN:62;ASN:62;HIS:64;HIS:94;HIS:96;HIS:119;THR:199;THR:200<br>Pi-cation: HIS:94
Scaposin	Keap-1	6T7V	-9.3	0.1	Hbond: VAL:463;VAL:465;LEU:557;THR:560;VAL:606<br>Hydrophobic: VAL:606
Scaposin	NFκB	4FA6	-7.3	0.4	Hbond: LYS:833;VAL:882<br>Hydrophobic: ILE:831
Scaposin	P38	1A9U	-7.2	1.6	Hbond: ASN:159<br>Hydrophobic: ASN:159;GLU:163<br>Pi-cation: LYS:165
Scaposin	PD-L1	5N2F	-9.3	0.9	Hbond: GLN:66;SER:117<br>Hydrophobic: ILE:54;TYR:56;ALA:121;TYR:123<br>Pi-stacking: TYR:56
Scaposin	Tert	5CQG	-8.1	1.0	Hbond: ARG:486;ARG:486;PHE:494;GLY:553;LEU:554<br>Hydrophobic: PHE:494;ILE:550;TYR:551<br>Pi-stacking: PHE:494
P-hydroxybenzoic acid rutinoside	AChE	7E3H	-7.6	0.6	Hbond: GLY:120;TYR:124;TYR:133;TYR:133;GLU:202;TYR:337<br>Hydrophobic: TRP:86
P-hydroxybenzoic acid rutinoside	AKT	4GV1	-7.2	0.5	Hbond: LYS:179;GLU:228;ALA:230;ALA:230;GLU:234;GLU:278;ASP:292;ASP:292<br>Hydrophobic: THR:211;MET:227;THR:291
P-hydroxybenzoic acid rutinoside	Amylase	2QV4	-7.5	0.7	Hbond: TYR:151;ARG:195;LYS:200;LYS:200;HIS:201;GLU:233;ILE:235;HIS:299<br>Hydrophobic: LEU:165
P-hydroxybenzoi	BChE	6EQP	-7.9	0.6	Hbond: ASP:70;GLY:115;GLY:115;TYR:128;TYR:128;TYR:332;HIS:438<br>Hydrophobic: TRP:82

c acid rutinoside					
P-hydroxybenzoic acid rutinoside	CDK2	6GUE	-7.3	0.8	Hbond: TYR:180;LEU:83;LEU:83;HIS:84;ASP:145<br>Hydrophobic: VAL:163<br>Salt bridge: ARG:50;ARG:126;ARG:150
P-hydroxybenzoic acid rutinoside	CDK4	2W96	-7.2	4.4	Hbond: ILE:12;LYS:35;GLU:94;HIS:95;VAL:96;VAL:96;VAL:96;ARG:101;ASP:158<br>Hydrophobic: PHE:93;ALA:157<br>Salt bridge: LYS:35;LYS:35
P-hydroxybenzoic acid rutinoside	Glucosidase	7KBJ	-7.0	0.6	Hbond: GLU:248;GLY:253;LYS:283;GLU:287;SER:290;SER:290;ARG:352;ARG:352<br>Hydrophobic: LEU:252<br>Salt bridge: ARG:352;ARG:352
P-hydroxybenzoic acid rutinoside	hCA I	3LXE	-7.7	0.9	Hbond: HIS:64;HIS:67;GLN:92;GLN:92;HIS:119;THR:199;THR:199;THR:199<br>Hydrophobic: ALA:121;LEU:198<br>Salt bridge: HIS:94;HIS:94
P-hydroxybenzoic acid rutinoside	hCA II	4IWZ	-7.2	0.5	Hbond: ASN:62;HIS:64;ASN:67;GLN:92;HIS:94;HIS:96;HIS:119;THR:199;THR:199<br>Salt bridge: HIS:94;HIS:94
P-hydroxybenzoic acid rutinoside	Keap-1	6T7V	-8.9	0.8	Hbond: LEU:365;ARG:415;ILE:416;VAL:418;GLY:462;VAL:463;VAL:463;VAL:465;VAL:465;VAL:604
P-hydroxybenzoic acid rutinoside	PD-L1	5N2F	-7.1	nd	Hbond: TYR:56;ILE:116;SER:117;ALA:121;ALA:121;ASP:122;ASP:122<br>Hydrophobic: ILE:54;TYR:56
Orobanchoside	AKT	4GV1	-9.1	0.7	Hbond: THR:160;LYS:163;LYS:179;ALA:230;ALA:230;ASP:274;LYS:276;ASN:279;ASN:279;THR:291;THR:291<br>Hydrophobic: LEU:156;PHE:161;PHE:161;VAL:164;PHE:438
Orobanchoside	Amylase	2QV4	-8.8	1.4	Hbond: TYR:62;GLN:63;ALA:198;LYS:200;HIS:201;GLU:233<br>Hydrophobic: TRP:59;TRP:59;TYR:62;LYS:200;LYS:200;ILE:235;ILE:235<br>Pi-stacking: TRP:59;HIS:201
Orobanchoside	BChE	6EQP	-9.5	6.0	Hbond: ASP:70;TRP:82;TRP:82;GLY:117;THR:120;GLU:197;TYR:332<br>Hydrophobic: TRP:82;LEU:286;ALA:328;PHE:329<br>Pi-stacking: TRP:82;PHE:329
Orobanchoside	BCL2	6O0K	-7.6	0.7	Hbond: ASP:111;GLU:136;ASN:143;ASN:143;ARG:146;ARG:146<br>Hydrophobic: PHE:104;VAL:133;LEU:137;ALA:149
Orobanchoside	Casp-3	3GJQ	-7.8	2.2	Hbond: ARG:207;ARG:207;ARG:207;ARG:207;ARG:207;ARG:207;ASN:208;SER:209;TRP:214;PHE:250;PHE:250;PHE:250<br>Hydrophobic: TRP:206;TRP:206;PHE:250;PHE:256
Orobanchoside	CDK2	6GUE	-8.9	8.1	Hbond: TYR:180;LYS:9;ILE:10;GLU:12;GLU:12;TYR:15;TYR:15;GLU:81;LEU:83;LEU:83;ASP:86<br>Hydrophobic: VAL:163;GLU:12;VAL:18;VAL:18;ALA:31;VAL:64;PHE:80;PHE:80;LEU:134;LEU:134<br>Salt bridge: ARG:50;ARG:126;ARG:150
Orobanchoside	CDK4	2W96	-8.4	6.4	Hbond: VAL:14;GLY:15;ALA:16;LYS:35;ASP:99;ASP:99;ARG:101;ARG:101;GLU:144;GLU:144;GLU:144;ASN:145;ASP:158<br>Hydrophobic: LEU:147;THR:177<br>Salt bridge: LYS:35;LYS:142

Orobanchoside	Cyclin D1	2W99	-7.9	0.5	Hbond: CYS:68;CYS:73;GLU:75;GLU:75;GLU:75;GLU:75;GLU:75;PHE:78;GLN:183;GLN:183;THR:184<br>Hydrophobic: LEU:65;GLU:69;LYS:72;PHE:78;ARG:179;LYS:180<br>Salt bridge: LYS:180
Orobanchoside	Glucosidase	7KBJ	-7.9	4.3	Hbond: GLN:247;GLY:253;LYS:283;GLU:287;GLU:287;LEU:344;ALA:345;ARG:352;ARG:352<br>Hydrophobic: LEU:252;TYR:291;LEU:344;PHE:350<br>Salt bridge: ARG:352
Orobanchoside	hCA I	3LXE	-8.9	6.5	Hbond: TYR:7;HIS:64;SER:65;PHE:66;HIS:67;GLN:92;GLN:92;HIS:119;ARG:173;THR:199;THR:199;THR:199;HIS:200<br>Hydrophobic: TRP:5;VAL:62;ALA:121;VAL:143;VAL:143;LEU:198;HIS:200;TRP:209<br>Pi-stacking: HIS:94<br>Salt bridge: HIS:64;HIS:67;HIS:94
Orobanchoside	hCA II	4IWZ	-8.6	4.9	Hbond: HIS:4;TRP:5;TRP:5;ASN:62;ASN:67;GLU:69;GLN:92;HIS:119;THR:199;THR:199;THR:200;THR:200;PRO:201<br>Hydrophobic: LEU:60;ASN:62;VAL:121;VAL:143;LEU:198<br>Pi-stacking: HIS:94<br>Salt bridge: HIS:64;HIS:94
Orobanchoside	Keap-1	6T7V	-10.2	0.6	Hbond: ILE:416;VAL:418;VAL:465;VAL:465;VAL:512;VAL:514;ILE:559;THR:560;VAL:561;VAL:561;VAL:606;VAL:606;VAL:608<br>Hydrophobic: VAL:369;ALA:607
Orobanchoside	NFκB	4FA6	-8.8	0.4	Hbond: SER:806;LYS:807;LYS:833;VAL:882;THR:887;THR:887;ASP:950;ASP:950;ASN:951;ASP:964<br>Hydrophobic: TRP:812;ILE:831;ILE:879;ILE:963;ILE:963;ILE:963<br>Salt bridge: LYS:833
Orobanchoside	P38	1A9U	-8.4	1.2	Hbond: LYS:66;ARG:70;ILE:147;ARG:149;ALA:172;ALA:172;HIS:174;THR:175;TYR:200;TYR:200;GLN:325;GLU:328;GLU:328<br>Hydrophobic: ILE:63;ILE:63;ARG:67;ILE:147;HIS:174<br>Salt bridge: LYS:66;ARG:70;ARG:149;HIS:174<br>Pi-cation: ARG:149
Orobanchoside	PD-L1	5N2F	-9.2	2.2	Hbond: THR:20;TYR:56;TYR:56;ASP:61;ASN:63;ASN:63;GLN:66;GLN:66;ASP:122;LYS:124;ARG:125<br>Hydrophobic: ILE:54;TYR:56;VAL:76;ALA:121;TYR:123<br>Salt bridge: LYS:75
Orobanchoside	Tert	5CQG	-7.8	57.4	Hbond: PHE:494;TRP:498;ILE:550;GLY:553;LEU:554<br>Hydrophobic: PHE:494<br>Pi-stacking: PHE:494;TYR:551<br>Salt bridge: ARG:557
Leucosceptoside B	AKT	4GV1	-9.4	1.6	Hbond: PHE:161;GLY:162;ASP:274;ASP:274;LYS:276;ASN:279;THR:291;ASP:292;ASP:292;GLY:294;ASP:439;ASP:439<br>Hydrophobic: LYS:179;PHE:442;PHE:442<br>Salt bridge: LYS:276<br>Pi-cation: LYS:179
Leucosceptoside B	Amylase	2QV4	-8.5	0.5	Hbond: ARG:195;ALA:198;HIS:201;GLU:233;GLU:233;ILE:235;HIS:299;ASP:300;HIS:305<br>Hydrophobic: TRP:59;TRP:59;LEU:162;THR:163;LEU:165;ILE:235<br>Pi-stacking: HIS:201
Leucosceptoside B	BChE	6EQP	-7.9	6.1	Hbond: ASP:70;GLY:78;SER:79;TRP:82;TRP:82;GLY:116;GLY:117;SER:198;ALA:199;TYR:332;TRP:430;TYR:440<br>Hydrophobic: ASP:70;TRP:82;TRP:82;THR:120;VAL:288<br>Pi-stacking: TRP:82<br>Salt bridge: HIS:438
Leucosceptoside B	BCL2	6O0K	-8.3	1.7	Hbond: GLU:136;GLU:136;GLU:136;ARG:139;ASN:143;ARG:146;ARG:146;ALA:149<br>Hydrophobic: PHE:104;TYR:108;THR:132;VAL:133;VAL:133;GLU:136;GLU:136;ASP:140
Leucosceptoside B	BRPF1	5MWZ	-7.2	1.8	Hbond: PHE:653;SER:654;SER:654;GLU:655;GLU:655;PRO:656;ILE:700<br>Hydrophobic: VAL:657;PRO:658;PRO:658;GLU:661<br>Pi-stacking: PHE:653
Leucosceptoside B	Casp-3	3GJQ	-7.7	8.1	Hbond: THR:62;THR:62;SER:65;SER:65;SER:65;SER:65;HIS:121;ARG:207;ARG:207;ARG:207;PHE:250<br>Hydrophobic: TRP:206;PHE:256

					Pi-stacking: PHE:256<br>Salt bridge: HIS:121;ARG:207
Leucosceptoside B	Casp-9	1JXQ	-7.2	1.2	Hbond: GLU:143;SER:144;ARG:146;ARG:146;ARG:146;GLY:147;PHE:399;PHE:400<br>Hydrophobic: LYS:401<br>Salt bridge: ARG:146;ARG:146
Leucosceptoside B	CDK2	6GUE	-8.4	5.3	Hbond: TYR:180;GLU:8;TYR:15;LYS:33;ASP:86;GLN:131;ASP:145<br>Hydrophobic: VAL:163;VAL:18;ALA:31;VAL:64;LYS:89;LEU:134;ALA:144<br>Pi-stacking: PHE:80<br>Salt bridge: ARG:50;ARG:126;ARG:150;LYS:89;LYS:89
Leucosceptoside B	CDK4	2W96	-8.5	8.6	Hbond: GLY:15;ALA:16;LYS:35;ASP:99;ASP:99;LYS:142;ASP:158;THR:177<br>Hydrophobic: ILE:12;ALA:33;GLU:144;LEU:147<br>Salt bridge: ARG:101
Leucosceptoside B	Cyclin D1	2W99	-7.4	6.9	Hbond: CYS:68;GLN:176;GLN:176;ARG:179;GLN:183;GLN:183<br>Hydrophobic: LYS:72;GLN:183<br>Salt bridge: LYS:180;LYS:180
Leucosceptoside B	hCA I	3LXE	-9.3	0.7	Hbond: SER:65;ASN:69;ASN:69;HIS:94;PHE:95;HIS:96;HIS:119;ARG:173;THR:199;THR:199;THR:199;HIS:200;HIS:200<br>Hydrophobic: ILE:60;VAL:62;VAL:143;VAL:143;LEU:198;TRP:209<br>Pi-stacking: HIS:94<br>Salt bridge: HIS:64;HIS:67;HIS:94;HIS:200
Leucosceptoside B	hCA II	4IWZ	-9.0	1.7	Hbond: ASN:62;ASN:62;ASN:62;HIS:64;ASN:67;ASN:67;GLN:92;HIS:94;HIS:96;HIS:119;THR:199;THR:200;THR:200;THR:200;PRO:201<br>Hydrophobic: ASN:62;VAL:121;VAL:121;PHE:131;VAL:143;LEU:198<br>Pi-stacking: HIS:94
Leucosceptoside B	Keap-1	6T7V	-9.1	1.5	Hbond: VAL:369;ILE:416;VAL:418;GLY:419;VAL:420;VAL:465;VAL:467;VAL:561;VAL:561<br>Hydrophobic: VAL:467;VAL:467;VAL:514;ILE:559;ALA:607;VAL:608
Leucosceptoside B	NFκB	4FA6	-8.5	11.4	Hbond: LYS:890;HIS:948;ASP:950;ASP:950;ASP:950;ASP:950;ASP:964<br>Hydrophobic: ILE:831;THR:887;ALA:889;ASP:950;ILE:963;ASP:964<br>Salt bridge: LYS:807;LYS:807;LYS:833
Leucosceptoside B	P38	1A9U	-8.1	3.1	Hbond: LYS:53;ARG:67;THR:106;SER:154;ASN:155;ASN:155;ASP:168;PHE:169;PHE:169;ARG:173;ARG:173<br>Hydrophobic: TYR:35;VAL:38;ALA:51;LYS:53;ILE:84;THR:106;ASP:168<br>Salt bridge: LYS:53;LYS:53;ARG:67;ARG:173
Leucosceptoside B	PD-L1	5N2F	-9.5	1.1	Hbond: ASN:63;GLN:66;ASP:73;LYS:75;LYS:75;LYS:75;LYS:75;TYR:123;ARG:125<br>Hydrophobic: ILE:54;TYR:56;TYR:56;LYS:75;VAL:76;ALA:121;TYR:123<br>Salt bridge: LYS:75;LYS:75;LYS:124
Leucosceptoside B	Tert	5CQG	-8.3	9.4	Hbond: ASN:192;ASN:192;ALA:195;ALA:195;ARG:253;ARG:253;ASP:254;ASP:254;TYR:256;GLN:308;ASP:343;ASN:369<br>Hydrophobic: PHE:193;PHE:193;TRP:302
Leucosceptoside A	AKT	4GV1	-10.3	0.8	Hbond: PHE:161;GLY:162;LYS:163;LYS:179;GLU:198;GLU:228;ALA:230;LYS:276;ASN:279;ASN:279;GLY:294<br>Hydrophobic: LEU:156;PHE:161;PHE:161;VAL:164;VAL:164;ALA:177;LEU:181;LEU:181;PHE:438
Leucosceptoside A	Amylase	2QV4	-9.0	1.1	Hbond: TRP:59;GLN:63;GLN:63;ALA:198;LYS:200;GLU:233;GLU:233;HIS:305<br>Hydrophobic: TRP:59;TRP:59;TYR:62;ILE:235<br>Pi-stacking: TRP:59;TYR:151<br>Salt bridge: HIS:305
Leucosceptoside A	BChE	6EQP	-10.5	0.3	Hbond: GLY:78;TRP:82;GLY:115;GLY:117;THR:120;THR:120;TYR:128;SER:198;TRP:430<br>Hydrophobic: TRP:82;TRP:82;LEU:286;ALA:328;PHE:329;TYR:332;PHE:398;TRP:430<br>Pi-stacking: TRP:231;TRP:231;PHE:329
Leucosceptoside A	BCL2	6O0K	-8.2	0.3	Hbond: ALA:100;ASP:111;GLU:136;GLU:136;ASP:140;ARG:146;ARG:146;ALA:149<br>Hydrophobic: PHE:104;TYR:108;ASP:111;LEU:137;ALA:149<br>Pi-stacking: PHE:104;PHE:104<br>Salt bridge: ARG:146

Leucosceptoside A	BRPF1	5MWZ	-7.5	5.8	Hbond: SER:654;SER:654;SER:654;GLU:655;GLU:655;PRO:656;ASN:703;ASN:708<br>Hydrophobic: PHE:653;VAL:657;PHE:714;PHE:714
Leucosceptoside A	Casp-3	3GJQ	-7.8	0.3	Hbond: THR:62;ARG:207;SER:209;SER:209;PHE:250<br>Hydrophobic: TRP:206;TRP:206;PHE:256;PHE:256<br>Pi-stacking: PHE:256<br>Salt bridge: ARG:207;ARG:207
Leucosceptoside A	CDK2	6GUE	-8.3	0.3	Hbond: TYR:180;ILE:10;GLU:12;TYR:15;GLU:81;ASP:86;GLN:131;ASN:132;ASN:132;ASP:145;ASP:145<br>Hydrophobic: VAL:163;ILE:10;TYR:15;VAL:18;VAL:18;ALA:31;LEU:134;LEU:134<br>Salt bridge: ARG:50;ARG:126;ARG:150<br>Pi-cation: LYS:129
Leucosceptoside A	CDK4	2W96	-8.6	1.2	Hbond: VAL:14;GLY:15;ALA:16;LYS:35;GLU:94;ARG:101;ARG:101;ASP:158;THR:177;THR:177<br>Hydrophobic: ILE:12;ALA:33;LYS:142;GLU:144;LEU:147;THR:177<br>Salt bridge: LYS:35;ARG:101;LYS:142
Leucosceptoside A	Cyclin D1	2W99	-7.3	0.5	Hbond: GLU:69;GLU:75;GLU:75;GLU:75;GLU:75;HIS:158;ASP:159;GLN:176<br>Hydrophobic: HIS:158;GLU:162;LYS:180;GLN:183<br>Salt bridge: ARG:179;LYS:180
Leucosceptoside A	Glucosidase	7KBJ	-7.5	0.7	Hbond: GLU:248;LEU:252;GLY:253;LYS:283;SER:290;SER:290;TYR:291;ARG:334;ALA:345;ARG:352;ARG:352<br>Hydrophobic: LEU:252;LEU:252;ILE:257;LEU:344;LEU:344;PHE:350<br>Salt bridge: ARG:352;ARG:352
Leucosceptoside A	hCA I	3LXE	-8.4	0.5	Hbond: HIS:67;GLN:92;GLN:92;HIS:94;HIS:96;THR:199;PRO:201<br>Hydrophobic: TRP:5;ALA:135;LEU:141<br>Pi-stacking: PHE:91<br>Salt bridge: HIS:64;HIS:67;HIS:200
Leucosceptoside A	hCA II	4IWZ	-8.3	0.9	Hbond: ASN:67;GLN:92;HIS:96;GLU:106;HIS:119;THR:199;THR:200;PRO:201<br>Hydrophobic: ASN:62;VAL:121;VAL:121;VAL:143;LEU:198<br>Pi-stacking: HIS:94<br>Salt bridge: HIS:64
Leucosceptoside A	Keap-1	6T7V	-8.7	1.0	Hbond: ARG:326;GLY:423;VAL:467;ALA:510;ALA:510;VAL:512;THR:560;VAL:561;VAL:606<br>Hydrophobic: VAL:420;VAL:420;ILE:559
Leucosceptoside A	NFκB	4FA6	-8.6	11.8	Hbond: ALA:805;SER:806;LYS:807;GLU:880;VAL:882;THR:887;THR:887;THR:887;LYS:890;ASP:950;ASN:951;ASP:964<br>Hydrophobic: ILE:831;ILE:879;ILE:963;ILE:963<br>Pi-stacking: TYR:867<br>Salt bridge: LYS:890
Leucosceptoside A	P38	1A9U	-7.8	13.6	Hbond: MET:109;ASP:112;ASP:112;ASN:155;PHE:169;PHE:169;PHE:169;ARG:173;ARG:173<br>Hydrophobic: VAL:30;ASP:168<br>Salt bridge: LYS:53
Leucosceptoside A	PD-L1	5N2F	-10.0	1.0	Hbond: ASN:63;GLN:66;VAL:68;ALA:121;TYR:123;LYS:124<br>Hydrophobic: ILE:54;TYR:56;TYR:56;VAL:68;MET:115;ALA:121;TYR:123
Leucosceptoside A	Tert	5CQG	-8.2	0.1	Hbond: LYS:189;ASN:192;ALA:195;ILE:252;ASP:254;ASP:254;ASP:254;ALA:255;TYR:256;GLN:308;ASN:369<br>Hydrophobic: LYS:189;PHE:193;PHE:193;TRP:302;ASP:343
Geniposide	AChE	7E3H	-8.7	1.4	Hbond: GLN:71;ASN:87;GLY:121;GLY:122;TYR:124;TYR:133<br>Hydrophobic: TYR:124;PHE:297;PHE:297;PHE:338
Geniposide	AKT	4GV1	-8.1	0.8	Hbond: PHE:161;GLY:162;LYS:179;GLU:191;HIS:194;GLU:198;ASP:274;LYS:276;PHE:293;GLY:294;THR:312<br>Hydrophobic: PHE:161;PHE:161;LEU:181
Geniposide	Amylase	2QV4	-8.0	0.5	Hbond: ARG:195;ARG:195;HIS:201;GLU:233;HIS:299<br>Hydrophobic: TRP:58;TYR:62;TYR:62
Geniposide	BChE	6EQP	-8.2	1.1	Hbond: ASP:70;GLY:116;GLY:117;SER:198;SER:198;SER:198;ALA:199;LEU:286<br>Hydrophobic: TRP:82

Geniposide	CDK2	6GUE	-8.1	0.8	Hbond: TYR:180;GLU:12;TYR:15;LYS:33;LEU:83;LEU:83;ASP:145<br>Hydrophobic: VAL:163;ASP:86;LEU:134<br>Salt bridge: ARG:50;ARG:126;ARG:150
Geniposide	CDK4	2W96	-8.4	0.8	Hbond: ALA:16;TYR:17;GLY:18;LYS:35;ASP:99;GLU:144;ASP:158;ASP:158;ASP:158<br>Hydrophobic: LEU:147;LEU:147<br>Salt bridge: LYS:35;LYS:35
Geniposide	Glucosidase	7KBJ	-7.8	0.9	Hbond: GLY:264;GLY:264;ASN:265;LYS:283;ARG:352<br>Hydrophobic: PHE:350;PHE:350<br>Salt bridge: ARG:334;ARG:352;ARG:352
Geniposide	hCA I	3LXE	-7.2	0.8	Hbond: GLN:92;HIS:94;HIS:96;THR:199;THR:199;THR:199;HIS:200<br>Hydrophobic: PHE:91;LEU:198
Geniposide	hCA II	4IWZ	-7.5	0.4	Hbond: ASN:67;GLN:92;HIS:94;HIS:96;HIS:119;THR:199<br>Hydrophobic: TRP:5;HIS:64<br>Salt bridge: HIS:64;HIS:94
Geniposide	Keap-1	6T7V	-8.5	1.0	Hbond: GLY:367;GLY:367;VAL:418;VAL:465;ALA:466;VAL:606<br>Hydrophobic: ILE:559
Geniposide	NFκB	4FA6	-7.1	0.8	Hbond: SER:806;THR:887;THR:887;ASP:950;ASN:951;ASP:964<br>Hydrophobic: LYS:833;ILE:879;ILE:963
Geniposide	PD-L1	5N2F	-7.5	3.5	Hbond: PHE:19;GLN:66;ASP:73;ALA:121<br>Hydrophobic: TYR:56;TYR:56;TYR:56;TYR:123<br>Salt bridge: LYS:124
Geniposide	Tert	5CQG	-7.5	0.4	Hbond: PHE:494;TRP:498;LEU:554<br>Hydrophobic: PHE:494;PHE:494;ILE:550;TYR:551
Eriodictyol hexoside	AChE	7E3H	-7.3	0.5	Hbond: TYR:72;ASP:74;ASN:87;TYR:124;TYR:124;GLY:126;GLU:202;TYR:337<br>Hydrophobic: TRP:86;TYR:337;TYR:337;PHE:338;PHE:338
Eriodictyol hexoside	AKT	4GV1	-9.2	1.1	Hbond: PHE:161;GLY:162;GLY:162;GLU:228;ALA:230;GLU:234<br>Hydrophobic: VAL:164;PHE:438
Eriodictyol hexoside	Amylase	2QV4	-9.4	1.0	Hbond: GLN:63;ASN:105;ALA:106;GLU:233;HIS:299<br>Hydrophobic: TRP:58;TRP:59;TRP:59;TYR:62;LEU:165
Eriodictyol hexoside	BChE	6EQP	-9.2	0.6	Hbond: GLY:115;GLY:117;GLY:117;TYR:128;GLU:197;SER:287<br>Hydrophobic: TRP:82;TRP:82;TRP:82;PHE:329
Eriodictyol hexoside	BCL2	6O0K	-8.0	9.1	Hbond: ASP:111;GLU:136;GLU:136;GLU:136;ASP:140;ASP:140<br>Hydrophobic: PHE:104;ASP:111;ALA:149;PHE:153<br>Salt bridge: ARG:146
Eriodictyol hexoside	BRPF1	5MWZ	-7.5	1.1	Hbond: ASN:651;SER:654;SER:654;SER:654;GLU:655;ASN:708;ASN:708<br>Hydrophobic: VAL:657;VAL:657;PRO:658;PHE:714
Eriodictyol hexoside	Casp-3	3GJQ	-8.1	1.0	Hbond: SER:63;SER:65;SER:65;HIS:121;GLY:122;GLY:122;ARG:207;ARG:207;SER:209<br>Salt bridge: ARG:207
Eriodictyol hexoside	Casp-9	1JXQ	-7.4	1.1	Hbond: GLU:143;ARG:146;ARG:146;PHE:399;THR:402;THR:402<br>Hydrophobic: ARG:146;LYS:401<br>Pi-cation: ARG:146
Eriodictyol hexoside	CDK2	6GUE	-9.2	0.9	Hbond: TYR:180;GLU:12;TYR:15;TYR:15;LEU:83;LYS:89;LYS:129;ASN:132;ASN:132;ASP:145<br>Hydrophobic: VAL:163;ILE:10;ILE:10;VAL:18;GLN:85;LYS:89;LEU:134<br>Salt bridge: ARG:50;ARG:126;ARG:150
Eriodictyol hexoside	CDK4	2W96	-9.5	0.8	Hbond: ALA:16;VAL:96;ASP:99;ASP:99;THR:102;THR:102;THR:102;LYS:142;ASN:145;ASP:158<br>Hydrophobic: ASP:99;GLU:144<br>Salt bridge: ARG:101
Eriodictyol hexoside	Cyclin D1	2W99	-7.9	1.0	Hbond: CYS:73;CYS:73;GLU:75;PHE:78;ALA:187;THR:191;GLN:261<br>Hydrophobic: LEU:65;ALA:187;LEU:188

Eriodictyol hexoside	Glucosidase	7KBJ	-9.8	1.1	Hbond: GLU:248;LEU:252;GLY:253;GLY:264;LYS:283;GLU:287;TYR:291;ARG:334;ALA:345;ARG:352<br>Hydrophobic: PHE:350<br>Pi-cation: ARG:352
Eriodictyol hexoside	hCA I	3LXE	-8.6	36.2	Hbond: TRP:5;TRP:5;ASP:8;ASN:11;VAL:233;ASP:236;GLN:242;HIS:243;HIS:243;HIS:243
Eriodictyol hexoside	hCA II	4IWZ	-8.1	0.9	Hbond: HIS:4;ASN:62;ASN:62;HIS:64;HIS:119;THR:199<br>Hydrophobic: VAL:121;LEU:198;THR:200<br>Pi-cation: HIS:94
Eriodictyol hexoside	Keap-1	6T7V	-10.1	0.5	Hbond: VAL:418;VAL:418;VAL:465;VAL:465;THR:560;GLY:603;VAL:604;VAL:606;VAL:606;VAL:608<br>Hydrophobic: ILE:559
Eriodictyol hexoside	NFκB	4FA6	-9.0	1.0	Hbond: LYS:807;LYS:808;LYS:833;VAL:882;VAL:882;LYS:890;ASP:950<br>Hydrophobic: TRP:812;ILE:831;ILE:879;ILE:963<br>Salt bridge: LYS:833
Eriodictyol hexoside	Nras	8VM2	-7.3	0.6	Hbond: ARG:97;LYS:135;GLN:165;TYR:166;LYS:169<br>Hydrophobic: PRO:110;ILE:139;GLU:162;TYR:166
Eriodictyol hexoside	P38	1A9U	-9.1	2.0	Hbond: ALA:34;SER:56;SER:56;HIS:64;ARG:67;PHE:169;ARG:173<br>Hydrophobic: TYR:35;ALA:172;ARG:173<br>Salt bridge: HIS:64;ARG:67
Eriodictyol hexoside	PD-L1	5N2F	-10.0	0.8	Hbond: ASP:61;ASN:63;GLN:66;TYR:123;TYR:123;TYR:123;LYS:124;ARG:125;ARG:125<br>Hydrophobic: TYR:56;TYR:56;MET:115;ALA:121;TYR:123<br>Salt bridge: LYS:124
Eriodictyol hexoside	Tert	5CQG	-8.2	49.5	Hbond: ARG:486;ASN:492;GLY:495;ILE:497;TRP:498;ARG:557;ARG:557<br>Hydrophobic: PHE:494;PHE:494;ILE:497;ILE:550;ILE:550;LEU:554<br>Salt bridge: ARG:557
Epicatechin	AChE	7E3H	-9.7	1.0	Hbond: TYR:72;TYR:72;ASP:74;GLY:120;TYR:133;GLU:202;TYR:337;TYR:341<br>Hydrophobic: ASP:74;TRP:86;TYR:124<br>Pi-stacking: TRP:86;TRP:86
Epicatechin	AKT	4GV1	-7.9	0.0	Hbond: LEU:156;LYS:158;LYS:158;LYS:158;GLU:228;ALA:230<br>Hydrophobic: LEU:156;VAL:164;ALA:177;THR:291;PHE:438
Epicatechin	Amylase	2QV4	-8.9	0.6	Hbond: GLN:63;ARG:195;ARG:195;ASP:197;ASP:300;HIS:305<br>Hydrophobic: TRP:58;TRP:59;TRP:59<br>Pi-stacking: TYR:62
Epicatechin	BChE	6EQP	-8.8	0.5	Hbond: GLY:115;THR:120;GLU:197<br>Hydrophobic: TRP:82;TRP:430<br>Pi-stacking: TRP:82;TRP:82
Epicatechin	BCL2	6O0K	-7.0	0.6	Hbond: ALA:100;ASP:103;ARG:107;ASN:143;ARG:146;ARG:146<br>Hydrophobic: PHE:104;PHE:104;TYR:108;ARG:146;VAL:148<br>Pi-stacking: PHE:104
Epicatechin	CDK2	6GUE	-8.7	0.0	Hbond: TYR:180;LYS:33;HIS:84;ASP:145;ASP:145<br>Hydrophobic: VAL:163;ILE:10;PHE:80;LEU:134;LEU:134;ALA:144<br>Salt bridge: ARG:50;ARG:126;ARG:150
Epicatechin	CDK4	2W96	-8.9	0.5	Hbond: ALA:16;LYS:35;VAL:96;ASP:99;ASP:99;ASP:99;LYS:142;ASN:145;ASP:158<br>Hydrophobic: ASP:99;GLU:144;LEU:147
Epicatechin	Cyclin D1	2W99	-7.0	1.0	Hbond: GLU:69;CYS:73;HIS:158;GLN:183<br>Hydrophobic: LEU:65;GLU:69;GLU:75;PHE:78
Epicatechin	Glucosidase	7KBJ	-8.4	0.5	Hbond: MET:289;TYR:291;TYR:291;ARG:334;ARG:352<br>Hydrophobic: LEU:252;ILE:257;TYR:291
Epicatechin	hCA I	3LXE	-7.7	0.4	Hbond: TYR:7;HIS:64;SER:65;GLN:92;THR:199;HIS:200<br>Hydrophobic: PHE:91;LEU:198;HIS:200

					Pi-stacking: HIS:94<br>Pi-cation: HIS:94
Epicatechin	hCA II	4IWZ	-7.7	0.8	Hbond: HIS:64;HIS:96;HIS:119;THR:199;THR:199<br>Hydrophobic: VAL:121;LEU:198<br>Pi-cation: HIS:94
Epicatechin	Keap-1	6T7V	-9.2	1.0	Hbond: GLY:367;GLY:462<br>Hydrophobic: ALA:366;ILE:559
Epicatechin	NFκB	4FA6	-7.5	1.1	Hbond: SER:806;GLU:880;VAL:882;VAL:882;ASN:951;ASP:964;ASP:964;ASP:964<br>Hydrophobic: PRO:810;LYS:833;ILE:879;VAL:882;ILE:963;ILE:963;ASP:964
Epicatechin	Nras	8VM2	-7.2	0.5	Hbond: ARG:97;VAL:109;MET:111;GLN:165;TYR:166;LYS:169<br>Hydrophobic: PRO:110;ILE:139;GLU:162;TYR:166
Epicatechin	P38	1A9U	-8.0	1.0	Hbond: GLU:81;ALA:157;ASN:159;ASN:159;LEU:164<br>Hydrophobic: ASN:159;GLU:163;LYS:165<br>Pi-cation: LYS:165
Epicatechin	PD-L1	5N2F	-9.3	1.1	Hbond: GLN:66;MET:115;MET:115;ASP:122;TYR:123<br>Hydrophobic: ILE:54;TYR:56;MET:115;MET:115;ALA:121;TYR:123<br>Pi-stacking: TYR:56
Epicatechin	Tert	5CQG	-8.8	0.5	Hbond: MET:482;ARG:486;TRP:498<br>Hydrophobic: PHE:494;ILE:497;TYR:551;LEU:554<br>Pi-stacking: PHE:494;PHE:494
Caffeic acid-4-O-glucuronide	BChE	6EQP	-8.3	0.9	Hbond: GLY:115;GLY:115;GLY:115;TYR:128;LEU:286<br>Hydrophobic: PHE:329
Caffeic acid-4-O-glucuronide	Keap-1	6T7V	-9.4	1.1	Hbond: GLY:367;GLY:462;VAL:463;VAL:463;VAL:514;ALA:556;LEU:557;THR:560;VAL:561<br>Salt bridge: ARG:415
Caffeic acid-3-O-glucuronide	AChE	7E3H	-9.1	1.0	Hbond: TRP:86;TRP:86;TYR:124;PHE:295;HIS:447<br>Hydrophobic: PHE:338<br>Pi-stacking: TYR:337
Caffeic acid-3-O-glucuronide	Keap-1	6T7V	-9.3	0.5	Hbond: GLY:367;VAL:418;GLY:462;VAL:465;VAL:465;THR:560;VAL:606<br>Hydrophobic: ALA:366
Caffeic acid pentosyl hexouronide	AKT	4GV1	-9.1	0.7	Hbond: LYS:158;LYS:179;ALA:230;ASP:274;GLY:294;LEU:295<br>Hydrophobic: VAL:164;VAL:164;ALA:177<br>Salt bridge: LYS:179
Caffeic acid pentosyl hexouronide	Amylase	2QV4	-7.9	0.6	Hbond: THR:163;LYS:200;HIS:201;GLU:240;HIS:299;ASP:300<br>Hydrophobic: TYR:151;TYR:151;TYR:151
Caffeic acid pentosyl hexouronide	BChE	6EQP	-8.3	7.2	Hbond: ASP:70;ASP:70;SER:79;TRP:82;GLY:115;GLY:115;GLY:115;GLY:121;ALA:328;HIS:438;HIS:438<br>Hydrophobic: ASP:70;TRP:82;THR:120
Caffeic acid pentosyl hexouronide	Casp-3	3GJQ	-7.7	7.9	Hbond: ARG:207;ARG:207;PHE:250;SER:251;PHE:252;ASP:253<br>Hydrophobic: TRP:206;ASP:253;PHE:256;PHE:256<br>Pi-stacking: PHE:256
Caffeic acid pentosyl hexouronide	CDK2	6GUE	-8.4	1.6	Hbond: TYR:180;GLU:12;TYR:15;LYS:33;ASP:127;LYS:129;GLN:131;ASN:132;ASN:132<br>Hydrophobic: VAL:163;ILE:10;ILE:10<br>Salt bridge: ARG:50;ARG:126;ARG:150;LYS:89;LYS:129
Caffeic acid pentosyl hexouronide	CDK4	2W96	-7.7	4.7	Hbond: VAL:14;GLY:15;ASP:99;ASP:99;ASP:158;ASP:158<br>Hydrophobic: ILE:12;LEU:147;LEU:147;ALA:157<br>Salt bridge: LYS:35;ARG:101;ARG:101

Caffeic acid pentosyl hexouronide	Cyclin D1	2W99	-7.5	0.9	Hbond: CYS:73;HIS:158;LYS:180;GLN:183;GLN:261<br>Hydrophobic: ALA:187;LEU:188
Caffeic acid pentosyl hexouronide	Glucosidase	7KBJ	-7.8	10.5	Hbond: TYR:246;GLU:248;LEU:252;GLY:253;GLY:253;GLY:254;ARG:267;TYR:348;PHE:350;PHE:350<br>Hydrophobic: LEU:252<br>Salt bridge: LYS:283;ARG:352
Caffeic acid pentosyl hexouronide	hCA I	3LXE	-7.7	6.2	Hbond: HIS:64;HIS:67;GLN:92;HIS:94;HIS:96;HIS:119;THR:199;THR:199;PRO:201;TYR:204<br>Hydrophobic: PRO:202<br>Salt bridge: HIS:94;HIS:200
Caffeic acid pentosyl hexouronide	hCA II	4IWZ	-7.6	0.6	Hbond: TYR:7;ASN:62;ASN:62;ASN:67;PHE:70;GLN:92;HIS:94;HIS:96;THR:199<br>Hydrophobic: GLU:69;ILE:91;GLN:92<br>Salt bridge: HIS:94
Caffeic acid pentosyl hexouronide	Keap-1	6T7V	-9.8	4.6	Hbond: GLY:364;LEU:365;GLY:367;GLY:367;GLY:367;ARG:415;ILE:416;GLY:464;VAL:514;ILE:559;VAL:606
Caffeic acid pentosyl hexouronide	NFκB	4FA6	-7.7	0.7	Hbond: SER:806;LYS:807;LYS:833;VAL:882;THR:887;LYS:890;ASN:951;ASP:964;ASP:964<br>Hydrophobic: TRP:812;ILE:831;ILE:963;ILE:963<br>Salt bridge: LYS:807
Caffeic acid pentosyl hexouronide	P38	1A9U	-7.2	1.1	Hbond: ARG:49;GLU:81;LEU:108;LEU:108;LEU:108;GLY:110;ASN:159;ASN:159<br>Hydrophobic: GLU:81;GLU:163<br>Salt bridge: ARG:136;LYS:165
Caffeic acid pentosyl hexouronide	PD-L1	5N2F	-8.7	0.9	Hbond: PHE:19;PHE:19;TYR:56;ASN:63;ASN:63;GLN:66;GLN:66;ALA:121<br>Hydrophobic: TYR:56;MET:115;ALA:121;TYR:123<br>Pi-stacking: TYR:56
Caffeic acid-4-O-glucuronide	AChE	7E3H	-8.7	0.6	Hbond: GLY:120;TYR:124;TYR:133;PHE:295;HIS:447<br>Hydrophobic: PHE:297;PHE:338<br>Pi-stacking: TYR:337
Caffeic acid-4-O-glucuronide	AKT	4GV1	-7.8	1.1	Hbond: PHE:161;GLY:162;ASP:274;LYS:276;ASN:279<br>Hydrophobic: ASP:292<br>Salt bridge: LYS:179
Caffeic acid-3-O-glucuronide	AKT	4GV1	-7.5	0.7	Hbond: LYS:158;LYS:158;THR:195<br>Hydrophobic: PHE:161;LYS:179<br>Salt bridge: LYS:179
Caffeic acid-4-O-glucuronide	Amylase	2QV4	-8.2	0.1	Hbond: TRP:59;GLN:63;HIS:101;ASP:197;ILE:235<br>Hydrophobic: LEU:162;ILE:235<br>Salt bridge: LYS:200;HIS:201
Caffeic acid-3-O-glucuronide	Amylase	2QV4	-7.9	1.0	Hbond: HIS:101;ASP:197;ASP:197;ALA:198<br>Hydrophobic: TRP:59;TRP:59;TRP:59;LEU:165<br>Salt bridge: HIS:305
Caffeic acid-3-O-glucuronide	BChE	6EQP	-8.8	0.9	Hbond: TRP:82;GLY:117;GLU:197;SER:198;ALA:199;PRO:285;TRP:430;TYR:440<br>Hydrophobic: TRP:82
Caffeic acid-3-O-glucuronide	BCL2	6O0K	-7.3	1.1	Hbond: ASP:111;ASP:111;ASP:111;ASN:143;PHE:153<br>Hydrophobic: LEU:137;ARG:146<br>Pi-stacking: PHE:104;TYR:108<br>Salt bridge: ARG:146
Caffeic acid-3-O-glucuronide	Casp-3	3GJQ	-7.1	1.0	Hbond: THR:62;SER:65;SER:65;SER:205;ARG:207;SER:209<br>Hydrophobic: TYR:204;TRP:206;PHE:256<br>Salt bridge: ARG:207;ARG:207

Caffeic acid-3-O-glucuronide	CDK2	6GUE	-8.7	1.0	Hbond: TYR:180;LEU:83;ASP:86;ASP:86;ASP:86;ASP:145<br>Hydrophobic: VAL:163;ALA:31;PHE:80;PHE:80;LEU:134;ALA:144<br>Salt bridge: ARG:50;ARG:126;ARG:150;LYS:33
Caffeic acid-4-O-glucuronide	CDK2	6GUE	-7.7	0.9	Hbond: TYR:180;TYR:15;TYR:15;TYR:15;LYS:33;GLN:131;ASP:145;ASP:145<br>Hydrophobic: VAL:163;ILE:10;ILE:10;LEU:134<br>Salt bridge: ARG:50;ARG:126;ARG:150;LYS:33;LYS:89
Caffeic acid-3-O-glucuronide	CDK4	2W96	-8.3	0.1	Hbond: VAL:14;GLY:15;ALA:16;VAL:96;ASP:99;ARG:101;THR:102;GLU:144;ASP:158<br>Hydrophobic: ILE:12;LEU:147;LEU:147<br>Salt bridge: LYS:35;HIS:95;ARG:101;LYS:142
Caffeic acid-4-O-glucuronide	CDK4	2W96	-7.9	1.2	Hbond: ALA:16;HIS:95;VAL:96;ASP:97;ASP:99;ASP:99;ARG:101;ASP:158<br>Hydrophobic: VAL:20<br>Salt bridge: LYS:35;LYS:142
Caffeic acid-4-O-glucuronide	Cyclin D1	2W99	-7.5	1.0	Hbond: CYS:68;GLU:75;PHE:78;GLN:176;GLN:176;ARG:179;GLN:183;GLN:183;ALA:187<br>Hydrophobic: LYS:180<br>Salt bridge: HIS:158;LYS:180
Caffeic acid-3-O-glucuronide	Cyclin D1	2W99	-7.3	0.4	Hbond: CYS:73;GLN:183;GLN:183<br>Hydrophobic: LEU:65;GLU:69;GLU:75;ALA:187<br>Salt bridge: HIS:158
Caffeic acid-4-O-glucuronide	Glucosidase	7KBJ	-7.9	0.2	Hbond: GLN:247;GLN:247;GLY:253;GLY:266;LYS:283;SER:290;SER:290;TYR:291;TYR:291<br>Hydrophobic: TYR:291;TYR:291<br>Salt bridge: ARG:334<br>Pi-cation: ARG:352
Caffeic acid-3-O-glucuronide	Glucosidase	7KBJ	-7.7	1.1	Hbond: GLN:247;LYS:283;GLU:287;TYR:291<br>Salt bridge: LYS:283;ARG:334;ARG:352;ARG:352<br>Pi-cation: ARG:352
Caffeic acid-4-O-glucuronide	hCA I	3LXE	-7.5	1.1	Hbond: GLN:92;HIS:94;HIS:96;HIS:119;THR:199;THR:199;THR:199<br>Hydrophobic: LEU:131;ALA:135;LEU:141;LEU:198<br>Pi-stacking: PHE:91<br>Salt bridge: HIS:64;HIS:94;HIS:94;HIS:96;HIS:200
Caffeic acid-3-O-glucuronide	hCA I	3LXE	-7.3	1.1	Hbond: TYR:7;HIS:64;GLN:92;HIS:94;HIS:96;THR:199;THR:199;HIS:200<br>Hydrophobic: PHE:91;LEU:141;LEU:198;LEU:198<br>Salt bridge: HIS:64;HIS:67;HIS:94;HIS:94
Caffeic acid-4-O-glucuronide	hCA II	4IWZ	-7.8	1.0	Hbond: TRP:5;ASN:62;HIS:64;THR:199;THR:200;PRO:201<br>Hydrophobic: VAL:121;PHE:131;LEU:198<br>Salt bridge: HIS:94;HIS:94;HIS:119
Caffeic acid-3-O-glucuronide	hCA II	4IWZ	-7.5	1.1	Hbond: TYR:7;GLY:63;GLU:239;LEU:240<br>Hydrophobic: PHE:231<br>Pi-stacking: PHE:231<br>Salt bridge: HIS:64;LYS:170
Caffeic acid-4-O-glucuronide	NFκB	4FA6	-7.9	0.5	Hbond: THR:887;THR:887;LYS:890;ASP:950;ASN:951;ASP:964<br>Hydrophobic: ILE:831;ILE:879;ILE:879;ILE:963;ASP:964
Caffeic acid-3-O-glucuronide	NFκB	4FA6	-7.7	3.8	Hbond: VAL:882;THR:887;ASP:950;ASP:950;ASP:964;ASP:964<br>Hydrophobic: TYR:867;ILE:879;ILE:963;ILE:963;ILE:963;ASP:964<br>Salt bridge: LYS:890
Caffeic acid-4-O-glucuronide	P38	1A9U	-7.2	15.3	Hbond: LYS:53;GLU:71;GLU:71;THR:106;MET:109;MET:109;ASP:168<br>Hydrophobic: LEU:75;ILE:84;LEU:104;THR:106<br>Salt bridge: LYS:53;LYS:53<br>Pi-cation: LYS:53

Caffeic acid-3-O-glucuronide	P38	1A9U	-7.0	1.5	Hbond: GLU:81;GLU:81;ASN:159;LEU:164<br>Hydrophobic: GLU:163<br>Salt bridge: LYS:165<br>Pi-cation: LYS:165
Caffeic acid-4-O-glucuronide	PD-L1	5N2F	-8.9	0.1	Hbond: ILE:54;ILE:54;GLN:66;SER:117;ASP:122<br>Hydrophobic: TYR:56;TYR:56;MET:115;TYR:123
Caffeic acid-3-O-glucuronide	PD-L1	5N2F	-8.6	0.2	Hbond: PHE:19;GLN:66;GLN:66;ALA:121<br>Hydrophobic: TYR:56;MET:115;ALA:121;TYR:123<br>Pi-stacking: TYR:56
Caffeic acid-4-O-glucuronide	Tert	5CQG	-8.1	1.1	Hbond: GLY:495;TRP:498;ILE:550;ARG:557<br>Hydrophobic: PHE:494;PHE:494;PHE:494;PHE:494;ILE:497;ILE:550;TYR:551;TYR:551;LEU:554
Caffeic acid-3-O-glucuronide	Tert	5CQG	-7.9	5.0	Hbond: TRP:498<br>Hydrophobic: PHE:494;PHE:494;ILE:550;TYR:551<br>Salt bridge: ARG:557
Apigenin	AChE	7E3H	-9.3	0.8	Hbond: TRP:86;TYR:133;GLU:202<br>Hydrophobic: TRP:86;TRP:86;PHE:297;PHE:338<br>Pi-stacking: TYR:341
Apigenin	AKT	4GV1	-7.8	0.1	Hbond: LYS:158;LYS:158;GLU:228;ALA:230;ALA:230<br>Hydrophobic: VAL:164;ALA:177
Apigenin	Amylase	2QV4	-9.0	1.0	Hbond: GLN:63;ARG:195;ARG:195;HIS:299;ASP:300<br>Hydrophobic: TRP:58;TRP:59;TYR:62<br>Pi-stacking: TRP:59;TRP:59;TYR:62
Apigenin	BChE	6EQP	-9.3	0.8	Hbond: TRP:82;GLU:197<br>Hydrophobic: TRP:82;TRP:82;TYR:332;TRP:430<br>Pi-stacking: TRP:82;TRP:82
Apigenin	BCL2	6O0K	-7.1	0.2	Hbond: ALA:100;ASN:143;ASN:143<br>Hydrophobic: PHE:104;PHE:104;TYR:108;ARG:146;VAL:148<br>Pi-stacking: PHE:104
Apigenin	Casp-3	3GJQ	-7.2	0.9	Hbond: HIS:121;SER:205;ARG:207;ARG:207;ARG:207<br>Hydrophobic: PHE:256<br>Pi-stacking: PHE:256
Apigenin	CDK2	6GUE	-9.1	0.9	Hbond: TYR:180;LYS:33;GLU:51;LEU:83;LEU:83;ASP:86;ASP:86;ASP:145<br>Hydrophobic: VAL:163;ILE:10;PHE:80;LEU:134;LEU:134;ALA:144;ASP:145<br>Salt bridge: ARG:50;ARG:126;ARG:150
Apigenin	CDK4	2W96	-8.6	0.5	Hbond: ALA:16;VAL:96;ASP:99;ASP:99;ASP:99;LYS:142<br>Hydrophobic: ASP:99;GLU:144;LEU:147
Apigenin	Glucosidase	7KBJ	-8.2	1.0	Hbond: GLN:247;TYR:291;ARG:334;ARG:352<br>Hydrophobic: ILE:257;TYR:291
Apigenin	hCA I	3LXE	-7.9	0.4	Hbond: HIS:64;SER:65;HIS:94;HIS:96;THR:199;THR:199;HIS:200<br>Hydrophobic: PHE:91;LEU:131;LEU:198;LEU:198;HIS:200<br>Pi-stacking: HIS:94<br>Pi-cation: HIS:200
Apigenin	hCA II	4IWZ	-7.9	0.6	Hbond: ASN:62;HIS:64;GLN:92;HIS:94;HIS:96;HIS:119;THR:199<br>Hydrophobic: TRP:5;HIS:64;THR:200<br>Pi-cation: HIS:94
Apigenin	Keap-1	6T7V	-8.7	0.2	Hbond: VAL:418;VAL:463;VAL:465;VAL:465;LEU:557;THR:560;THR:560;VAL:561<br>Hydrophobic: ILE:559
Apigenin	NFκB	4FA6	-7.8	0.8	Hbond: VAL:882;VAL:882;LYS:890<br>Hydrophobic: TRP:812;TYR:867;ILE:879;ILE:879;ILE:879;ILE:963;ILE:963;ASP:964

Apigenin	Nras	8VM2	-7.6	1.6	Hbond: ARG:97;GLN:165;TYR:166;LYS:169<br>Hydrophobic: PRO:110;TYR:166
Apigenin	P38	1A9U	-7.6	0.9	Hbond: GLU:81;ASN:159<br>Hydrophobic: ALA:157;GLU:163
Apigenin	PD-L1	5N2F	-9.7	1.1	Hbond: GLN:66;MET:115;SER:117;ALA:121<br>Hydrophobic: ILE:54;ILE:54;TYR:56;TYR:56;MET:115;ALA:121;TYR:123
Apigenin	PPARγ	1I7I	-7.0	0.1	Hydrophobic: ARG:288;ARG:288;ILE:341;ILE:341;ILE:341
Apigenin	Tert	5CQG	-8.7	1.0	Hbond: GLY:553<br>Hydrophobic: MET:482;ILE:497;ILE:550;TYR:551;LEU:554;LEU:554;LEU:554<br>Pi-stacking: PHE:494
Acteoside	AKT	4GV1	-9.5	2.6	Hbond: PHE:161;GLY:162;ALA:230;GLU:234;ASN:279;THR:291;ASP:292;ASP:439<br>Hydrophobic: VAL:164;VAL:164;PHE:236;THR:291;ASP:439;PHE:442
Acteoside	Amylase	2QV4	-8.9	0.6	Hbond: GLN:63;ARG:195;ARG:195;ASP:197;ALA:198;GLU:233;GLU:233;HIS:299<br>Hydrophobic: TRP:59;TRP:59;TYR:62;TYR:62;THR:163<br>Salt bridge: HIS:305
Acteoside	BChE	6EQP	-9.5	1.3	Hbond: ASP:70;TRP:82;TRP:82;ASN:83;GLY:116;GLY:117;PRO:285;HIS:438;HIS:438<br>Hydrophobic: ASP:70;TRP:82;TRP:82;TRP:82;THR:120;LEU:125;PHE:329<br>Salt bridge: HIS:438
Acteoside	BCL2	6O0K	-7.9	8.1	Hbond: ASP:103;ASP:103;ARG:107;ASP:111;ASP:111;ARG:146;ARG:146<br>Hydrophobic: PHE:104;PHE:104;PHE:104;TYR:108;ASP:111;LEU:137;VAL:148;ALA:149<br>Pi-stacking: PHE:104<br>Salt bridge: ARG:146
Acteoside	BRPF1	5MWZ	-7.5	8.6	Hbond: ILE:652;ILE:652;SER:654;SER:654;GLU:655;GLU:655;PRO:656;ASN:708;ASN:708<br>Hydrophobic: VAL:657;PRO:658;PRO:658;TYR:707
Acteoside	Casp-3	3GJQ	-8.0	1.4	Hbond: SER:205;ARG:207;ARG:207;ARG:207;GLU:248;PHE:250;PHE:250;PHE:252;ASP:253<br>Hydrophobic: TYR:204;TRP:206;PHE:250;PHE:256;PHE:256
Acteoside	CDK2	6GUE	-8.7	0.9	Hbond: TYR:180;GLU:12;TYR:15;LYS:33;ASP:127;ASP:127;ASN:132;ASN:132;ASP:145;ASP:145;ASP:145<br>Hydrophobic: VAL:163;TYR:15;VAL:18;ALA:31;VAL:64;PHE:80;PHE:80;GLN:131;LEU:134;ALA:144<br>Salt bridge: ARG:50;ARG:126;ARG:150<br>Pi-cation: LYS:129
Acteoside	CDK4	2W96	-8.8	9.0	Hbond: VAL:14;GLY:15;GLU:94;HIS:95;VAL:96;ASP:99;ARG:101;ARG:101;THR:102;GLU:144;GLU:144;THR:177<br>Hydrophobic: ASP:99;LEU:147;LEU:147;ALA:157<br>Salt bridge: ARG:101;ARG:101
Acteoside	Cyclin D1	2W99	-7.7	1.3	Hbond: LYS:72;HIS:158;GLN:176;LYS:180;GLN:183;GLN:183;ALA:187<br>Hydrophobic: LEU:65;GLU:69;GLU:75;PHE:78;PHE:78
Acteoside	hCA I	3LXE	-8.3	0.7	Hbond: SER:65;SER:65;HIS:96;GLU:106;HIS:119;THR:199;THR:199;THR:199;HIS:200<br>Hydrophobic: VAL:62;PHE:91;VAL:143;VAL:143;LEU:198;TRP:209<br>Pi-stacking: HIS:94<br>Salt bridge: HIS:67
Acteoside	hCA II	4IWZ	-8.8	9.6	Hbond: ASN:62;ASN:67;GLU:69;PHE:70;PHE:70;GLN:92;THR:199;THR:199;THR:199;THR:200;THR:200;THR:200<br>Hydrophobic: ALA:65;GLU:69;ILE:91;VAL:121;PHE:131;VAL:143;LEU:198<br>Pi-cation: HIS:94
Acteoside	Keap-1	6T7V	-9.0	1.8	Hbond: ARG:326;ARG:326;VAL:369;GLY:423;ALA:510;ALA:510;VAL:512;VAL:514<br>Hydrophobic: VAL:420;VAL:467;ILE:559
Acteoside	NFκB	4FA6	-8.5	0.5	Hbond: ALA:805;LYS:833;LYS:890;ASP:950;ASP:950;ASP:950;ASN:951;ASP:964;ASP:964;ASP:964;ASP:964<br>Hydrophobic: LYS:808;TYR:867;ILE:879;ILE:879;ILE:963<br>Salt bridge: LYS:890

Acteoside	Nras	8VM2	-7.1	3.8	Hbond: ASP:108;TYR:137;TYR:137;THR:158;GLU:162;GLU:162;GLN:165<br>Hydrophobic: ASP:108;GLU:162<br>Salt bridge: LYS:169
Acteoside	P38	1A9U	-7.9	1.8	Hbond: TYR:35;LYS:53;LYS:53;GLU:71;ASP:112;ASP:112;ASP:112;PHE:169<br>Hydrophobic: VAL:38;LYS:53;THR:106;MET:109;ASP:112;ALA:157;ASP:168
Acteoside	PD-L1	5N2F	-9.8	20.0	Hbond: PHE:19;PHE:19;ASN:63;GLN:66;GLN:66;VAL:68;GLU:71;GLU:71;ILE:116;ALA:121;ASP:122<br>Hydrophobic: ILE:54;TYR:56;VAL:68;ASP:73;MET:115;ALA:121
Acteoside	Tert	5CQG	-8.7	25.4	Hbond: ASN:192;ALA:195;ILE:252;ASP:254;GLN:308;GLN:308;ASN:369<br>Hydrophobic: LYS:189;LYS:189;PHE:193;PHE:193;TRP:302<br>Pi-cation: LYS:189
8-O-methylretusin	AChE	7E3H	-8.7	1.0	Hbond: GLY:120;TYR:124<br>Hydrophobic: TRP:86;PHE:338<br>Pi-stacking: TYR:337;TYR:341
8-O-methylretusin	AKT	4GV1	-8.0	1.0	Hbond: GLU:228;ALA:230<br>Hydrophobic: LEU:156;LEU:156;VAL:164;ALA:177;PHE:438
8-O-methylretusin	Amylase	2QV4	-8.6	0.9	Hbond: GLN:63;HIS:299<br>Hydrophobic: TRP:58;TYR:62;TYR:62<br>Pi-stacking: TRP:59
8-O-methylretusin	BChE	6EQP	-7.9	1.0	Hydrophobic: ALA:328<br>Pi-stacking: TRP:82;PHE:329
8-O-methylretusin	CDK2	6GUE	-8.9	0.8	Hbond: TYR:180;LYS:33;GLU:51;ASP:145;ASP:145<br>Hydrophobic: VAL:163;ILE:10;ALA:31<br>Pi-stacking: PHE:80<br>Salt bridge: ARG:50;ARG:126;ARG:150
8-O-methylretusin	CDK4	2W96	-8.6	0.9	Hbond: HIS:95;VAL:96;ARG:101;LYS:142<br>Hydrophobic: LYS:142;GLU:144;LEU:147
8-O-methylretusin	Glucosidase	7KBJ	-7.4	1.0	Hbond: LYS:283;ARG:334<br>Hydrophobic: LEU:252;ILE:257;TYR:291
8-O-methylretusin	hCA I	3LXE	-7.8	0.9	Hbond: HIS:94;HIS:96;HIS:119;THR:199;THR:199<br>Hydrophobic: LEU:131;LEU:198;TRP:209<br>Pi-stacking: HIS:94
8-O-methylretusin	hCA II	4IWZ	-7.3	19.8	Hbond: ASP:85;ASP:85;LYS:127;ASP:139<br>Hydrophobic: PRO:83;ASP:85;ASP:139;PRO:195;THR:208
8-O-methylretusin	Keap-1	6T7V	-8.3	1.0	Hbond: ARG:415;VAL:465<br>Hydrophobic: ALA:366;ILE:559
8-O-methylretusin	NFκB	4FA6	-7.7	1.1	Hbond: GLU:880;VAL:882<br>Hydrophobic: PRO:810;ILE:831;LYS:833;ILE:879;ILE:963;ASP:964<br>Pi-stacking: TYR:867
8-O-methylretusin	PD-L1	5N2F	-9.8	0.7	Hbond: GLN:66<br>Hydrophobic: ILE:54;TYR:56;TYR:56;MET:115;MET:115;ALA:121;ALA:121;TYR:123
8-O-methylretusin	PPARγ	1I7I	-7.1	0.8	Hbond: LYS:263<br>Hydrophobic: ARG:288;ARG:288;ILE:341;ILE:341;ILE:341
8-O-methylretusin	Tert	5CQG	-8.8	8.5	Hbond: ARG:486;ARG:486<br>Hydrophobic: MET:482;PHE:494;ILE:497;ILE:550;TYR:551;LEU:554;LEU:554<br>Pi-stacking: PHE:494;PHE:494
